# Risk of hospital admission with coronavirus disease 2019 in healthcare workers and their households: nationwide linkage cohort study

**DOI:** 10.1136/bmj.m3582

**Published:** 2020-10-28

**Authors:** Anoop S V Shah, Rachael Wood, Ciara Gribben, David Caldwell, Jennifer Bishop, Amanda Weir, Sharon Kennedy, Martin Reid, Alison Smith-Palmer, David Goldberg, Jim McMenamin, Colin Fischbacher, Chris Robertson, Sharon Hutchinson, Paul McKeigue, Helen Colhoun, David A McAllister

**Affiliations:** 1 Non-communicable Disease Epidemiology, London School of Hygiene and Tropical Medicine, London, UK; 2 Department of Cardiology, Imperial College NHS Trust, London, UK; 3 Public Health Scotland, Edinburgh, UK; 4 Centre for Population Health Sciences, University of Edinburgh, Edinburgh, UK; 5 School of Health and Life Sciences, Glasgow Caledonian University, Glasgow, UK; 6 MRC Institute of Genetics and Molecular Medicine, University of Edinburgh, Edinburgh, UK; 7 Usher Institute, University of Edinburgh, Edinburgh, UK; 8 Institute of Health and Wellbeing, University of Glasgow, 1 Lilybank Gardens, Glasgow G12 8RZ, UK

## Abstract

**Objective:**

To assess the risk of hospital admission for coronavirus disease 2019 (covid-19) among patient facing and non-patient facing healthcare workers and their household members.

**Design:**

Nationwide linkage cohort study.

**Setting:**

Scotland, UK, 1 March to 6 June 2020.

**Participants:**

Healthcare workers aged 18-65 years, their households, and other members of the general population.

**Main outcome measure:**

Admission to hospital with covid-19.

**Results:**

The cohort comprised 158 445 healthcare workers, most of them (90 733; 57.3%) being patient facing, and 229 905 household members. Of all hospital admissions for covid-19 in the working age population (18-65 year olds), 17.2% (360/2097) were in healthcare workers or their households. After adjustment for age, sex, ethnicity, socioeconomic deprivation, and comorbidity, the risk of admission due to covid-19 in non-patient facing healthcare workers and their households was similar to the risk in the general population (hazard ratio 0.81 (95% confidence interval 0.52 to 1.26) and 0.86 (0.49 to 1.51), respectively). In models adjusting for the same covariates, however, patient facing healthcare workers, compared with non-patient facing healthcare workers, were at higher risk (hazard ratio 3.30, 2.13 to 5.13), as were household members of patient facing healthcare workers (1.79, 1.10 to 2.91). After sub-division of patient facing healthcare workers into those who worked in “front door,” intensive care, and non-intensive care aerosol generating settings and other, those in front door roles were at higher risk (hazard ratio 2.09, 1.49 to 2.94). For most patient facing healthcare workers and their households, the estimated absolute risk of hospital admission with covid-19 was less than 0.5%, but it was 1% and above in older men with comorbidity.

**Conclusions:**

Healthcare workers and their households contributed a sixth of covid-19 cases admitted to hospital. Although the absolute risk of admission was low overall, patient facing healthcare workers and their household members had threefold and twofold increased risks of admission with covid-19.

## Introduction

Severe acute respiratory syndrome coronavirus 2 (SARS-CoV-2) continues to spread globally, with more than 8 million cases of coronavirus disease 2019 (covid-19) and more than half a million deaths as of 10 July 2020.^1^

Healthcare workers, who have been integral to the response to covid-19, may be at increased risk of contracting SARS-CoV-2 and hence subsequently transmitting it to their household, workplace contacts, or both.^2^ ^3^ Estimating the risk in this population is important to guide public health measures to protect healthcare workers and their families, maintain a functioning healthcare system, and control rates of secondary transmission within the community.^4^

Despite this, the extent of these risks is not well understood, as most studies have been in single centres and limited by small sample sizes and/or biased selection and recording of disease.^2^ ^5^ We are well placed to overcome these limitations in Scotland for two reasons. Firstly, the overwhelming majority of healthcare (especially acute care) is directly delivered by the National Health Service (NHS), which also maintains a national database on all directly employed staff in Scotland, including nursing, medical, and support staff and allied health professionals. Secondly, Scotland has a well established health record linkage system.^6^ ^7^ ^8^

Using record linkage, we evaluated the risk of admission to hospital with covid-19 among healthcare workers in patient and non-patient facing roles along with the risk in their household members. We further evaluated the risk of admission with covid-19 in patient facing healthcare workers in different clinical settings including intensive care and “front door” departments.

## Methods

### Population, data sources, and record linkage

We included healthcare workers if on 1 March 2020 (the date of the first positive reported case of covid-19 in Scotland) they were directly employed by the NHS, contracted to provide NHS general practice services in Scotland, or both. We defined healthcare workers as people providing healthcare services, whether they did so directly (for example, doctors and nurses) or indirectly (for example, laboratory technicians or people working with information systems).^9^ Healthcare workers’ data came from the Scottish Workforce Information Standard System (SWISS) and General Practitioner Contractor Database (GPCD) (appendix 1). We excluded dental staff and those working exclusively in paediatric roles, in addition to other exclusions due to incomplete or inconsistent data (appendix 2). Healthcare workers’ data were linked to the Community Health Index (CHI) database, a registry of all patients registered to receive care from the NHS in Scotland, close to the complete population. The CHI database includes individuals’ CHI number, a unique patient identifier used on all healthcare records in Scotland.

We used the CHI number to create a cohort linking these data on healthcare workers to multiple Scotland-wide databases (supplementary figure A). These included datasets containing individual level clinical information for virology testing for SARS-CoV-2, general hospital admission data, community prescribing, critical care admissions, and the national register for deaths (appendix 1).

We also used the CHI database to identify all individuals who were not themselves healthcare workers but shared a household with a healthcare worker. We assigned people to the same household if the address (including house and, if included, apartment number) on the CHI database was identical for both; fuzzy matching was not allowed. These household members were then also linked to the Scotland-wide datasets to construct a household member specific cohort (supplementary figure A). The healthcare worker cohort was restricted to the working age population (18-65 years), but the household member cohort included all ages.

Finally, we appended selected variables from the healthcare worker and household member data to an existing Scottish case-control study, REACT-COVID-19.^10^ REACT-COVID-19 included linked patient data (excluding healthcare worker and household member status) of all cases with a positive SARS-CoV-2 test or covid-19 as a cause of death on certification in Scotland. We matched each case to 10 age-sex geographically (general practice area) matched controls from the Scottish population. We used a nested case-control design, as this minimises the time needed for data processing and computation without loss of statistical power. These data allowed for comparisons with the general population, defined as residents of Scotland who were not healthcare workers or members of their households.

### Outcomes

We restricted outcomes to the time period from 1 March to 6 June 2020. The primary outcome was admission to hospital with covid-19, defined as the first positive test for SARS-CoV-2 in hospital and/or the individual being admitted within 28 days of testing positive. Secondary outcomes reported were admission to intensive care and death occurring within 28 days of first testing positive. We included tests irrespective of whether they were done for screening or clinical purposes. We chose hospital admission as the primary outcome because milder disease not requiring admission is likely to be subject to ascertainment bias (as healthcare workers may be more likely to be tested), and because admission with covid-19 is a clinically significant event.

### Exposure

We defined occupational roles for all healthcare workers by using the SWISS/GPCD databases. We categorised broad roles into patient facing, non-patient facing, or undetermined. We defined roles on the basis of formal job titles for nursing staff, allied health professionals, and support staff and specialty for medical staff. Selected nursing staff were additionally assigned on the basis of their working location (for example, the emergency department). We deliberately made these definitions narrow, assigning around a fifth of healthcare workers to “undetermined” (appendix 3). We did this to avoid non-differential misclassification bias. We assigned household members to the role of the associated member of staff (patient facing, non-patient facing, or undetermined). Where a household included more than more than one healthcare worker, we applied the highest risk designation.

We further divided patient facing roles into the following settings: “front door” (for example, paramedics or workers in acute receiving specialties), intensive care, non-intensive care but still exposed to aerosol generating procedures (for example, workers in respiratory medicine), and “other.” We made these designations before database linkage (see statistical analysis plan). During the course of the pandemic, guidance on infection prevention and control was updated. The key changes that took place, including the release dates, are summarised in appendix 4.

### Covariates

Occupation related covariates obtained from the healthcare worker database were seniority grade, occupation (medical, nursing, allied health professional, support, administration, and other), length of service, immigration status, and full/part time working status. We obtained age, sex, and fifth of the Scottish Index of Multiple Deprivation (SIMD), an area based measure of socioeconomic deprivation,^11^ from the CHI register. We identified comorbidities by using predefined criteria from previous hospital admissions (appendix 5), recently dispensed drugs, or both. Ethnicity was recorded across multiple datasets defined using the ONOMAP algorithm.^12^

### Missing data

A small proportion of people in the SWISS database who failed to meet the criteria for inclusion may have done so because of missing data (appendix 2). However, among those selected, no data were missing for the variables included in the regression model, other than part time status (which was not collected for general practitioners) and ethnicity. For ethnicity, missingness was caused when the ONOMAP algorithm failed to assign an ethnic group. As this was rare (1.22%), we used simple imputation to assign an ethnicity based on the most common ethnicity in the household or, where this was missing, for all members of a household in Scotland.

### Statistical analysis

We plotted the cumulative incidence of admission to hospital with covid-19 for healthcare workers, household members, and working age adults in the general population who were not healthcare workers or their household members. We obtained the denominator for the last group by subtracting the healthcare worker and household cohorts from the 2019 mid-year estimates. In the healthcare worker and household cohorts, we modelled hospital admission with covid-19 by using Cox regression, calculating robust standard errors to allow for clustering due to shared household membership and stratifying on groups of health board areas to allow for differences in baseline hazard. We chose these strata a priori on the basis of data for the general Scottish population. We treated age as a continuous covariate. To avoid residual confounding due to any non-linearity in the association between age and the (log) hazard rate, we fitted age by using a penalised spline function.

In the case-control study, we did conditional logistic regression. As REACT-COVID-19 used incidence density sampling,^10^ the effect measure estimates derived from these case-control analysis are directly comparable to those derived from the Cox regression. To allow comparison against the general population across the cohort and case-control analyses, we used the non-patient facing role as a common reference group.

We have also provided a separate prespecified statistical analysis plan. We used R version 3.6.1 for analyses. The analytical code is available at https://github.com/ChronicDiseaseEpi/hcw/.

### Patient and public involvement

No patients were involved in setting the research question or the outcome measures, nor were they involved in developing plans for design of the study. No patients were asked to advise on interpretation or writing up of results.

## Results

The cohort comprised 158 445 healthcare workers and 229 905 household members. Most healthcare workers (124 661; 78.7%), but only 88 274 (38.4%) household members, were women. More than half of healthcare workers (90 733; 57.3%) were patient facing, with 32 615 (20.6%) classified as non-patient facing and 35 097 (22.2%) as undetermined (table 1). Most patient facing healthcare workers were in “front door” roles (supplementary table A).

**Table 1:** Baseline characteristics of healthcare workers and members of their households. Values are numbers (percentages) unless stated otherwise

Characteristics	Healthcare workers					Household members of healthcare workers			
	Total (n=158 445)	Non-patient facing healthcare workers (n=32 615)	Undetermined healthcare workers (n=35 097)	Patient facing healthcare workers (n=90 733)		Total household members of healthcare workers (n=229 905)	Household of non-patient facing healthcare workers (n=44 812)	Household of undetermined healthcare workers (n=48 530)	Household of patient facing healthcare workers (n=136 563)
Mean (SD) age, years	44.49 (11.56)	46.64 (11.09)	45.7 (11.7)	43.24 (11.51)		30.88 (20.93)	33.19 (21.19)	31.86 (20.98)	29.77 (20.74)
Female sex	124 661 (78.7)	26 299 (80.6)	25 916 (73.8)	72 446 (79.8)		88 274 (38.4)	16 589 (37)	18 651 (38.4)	53 034 (38.8)
Fifth of SIMD:									
1 (least deprived)	24 066 (15.2)	4895 (15)	6773 (19.3)	12 398 (13.7)		31 337 (13.6)	5935 (13.2)	8510 (17.5)	16 892 (12.4)
2	29 894 (18.9)	6338 (19.4)	7100 (20.2)	16 456 (18.1)		41 466 (18)	8457 (18.9)	9533 (19.6)	23 476 (17.2)
3	31 213 (19.7)	6465 (19.8)	6570 (18.7)	18 178 (20)		44 291 (19.3)	8621 (19.2)	8735 (18)	26 935 (19.7)
4	35 528 (22.4)	7431 (22.8)	6970 (19.9)	21 127 (23.3)		53 197 (23.1)	10 456 (23.3)	10 117 (20.8)	32 624 (23.9)
5 (most deprived)	37 744 (23.8)	7486 (23)	7684 (21.9)	22 574 (24.9)		59 614 (25.9)	11 343 (25.3)	11 635 (24)	36 636 (26.8)
Ethnic group:									
White	153 126 (96.6)	31 991 (98.1)	33 813 (96.3)	87 322 (96.2)		219 914 (95.7)	43 445 (96.9)	46 163 (95.1)	130 306 (95.4)
South Asian	3704 (2.3)	453 (1.4)	899 (2.6)	2352 (2.6)		6600 (2.9)	919 (2.1)	1594 (3.3)	4087 (3)
Black	657 (0.4)	70 (0.2)	150 (0.4)	437 (0.5)		1385 (0.6)	157 (0.4)	309 (0.6)	919 (0.7)
Chinese	462 (0.3)	40 (0.1)	115 (0.3)	307 (0.3)		672 (0.3)	89 (0.2)	170 (0.4)	413 (0.3)
Other	496 (0.3)	61 (0.2)	120 (0.3)	315 (0.3)		1334 (0.6)	202 (0.5)	294 (0.6)	838 (0.6)
Comorbidity:	21 143 (13.3)	4701 (14.4)	4864 (13.9)	11 578 (12.8)		20 978 (9.1)	4599 (10.3)	4867 (10)	11 512 (8.4)
Ischaemic heart disease	1614 (1.0)	338 (1.0)	461 (1.3)	815 (0.9)		2798 (13.3)	634 (13.8)	676 (13.9)	1488 (12.9)
Other heart disease	3726 (2.4)	812 (2.5)	854 (2.4)	2060 (2.3)		4577 (21.8)	1037 (22.5)	1063 (21.8)	2477 (21.5)
Other circulatory system diseases	2450 (1.5)	487 (1.5)	593 (1.7)	1370 (1.5)		2967 (14.1)	644 (14)	721 (14.8)	1602 (13.9)
Advanced chronic kidney disease	110 (0.1)	41 (0.1)	19 (0.1)	50 (0.1)		160 (0.8)	26 (0.6)	42 (0.9)	92 (0.8)
Asthma and chronic lower respiratory disease	3349 (2.1)	705 (2.2)	771 (2.2)	1873 (2.1)		4169 (19.9)	861 (18.7)	965 (19.8)	2343 (20.4)
Neurological disorders	691 (0.4)	173 (0.5)	129 (0.4)	389 (0.4)		1011 (4.8)	216 (4.7)	229 (4.7)	566 (4.9)
Severe liver disease	96 (0.1)	15 (0)	27 (0.1)	54 (0.1)		134 (0.6)	29 (0.6)	29 (0.6)	76 (0.7)
Malignant neoplasms	5662 (3.6)	1323 (4.1)	1269 (3.6)	3070 (3.4)		3880 (18.5)	853 (18.5)	898 (18.5)	2129 (18.5)
Disorders of oesophagus, stomach, and duodenum	3886 (2.5)	872 (2.7)	854 (2.4)	2160 (2.4)		3496 (16.7)	776 (16.9)	787 (16.2)	1933 (16.8)
Diabetes, type 1	1080 (0.7)	249 (0.8)	227 (0.6)	604 (0.7)		1204 (5.7)	250 (5.4)	234 (4.8)	720 (6.3)
Diabetes, type 2	3763 (2.4)	944 (2.9)	972 (2.8)	1847 (2)		4762 (22.7)	1093 (23.8)	1210 (24.9)	2459 (21.4)
Diabetes, type unknown	284 (0.2)	80 (0.2)	58 (0.2)	146 (0.2)		302 (0.1)	72 (0.2)	72 (0.1)	158 (0.1)
Comorbidity count:									
0	137 302 (86.7)	27 914 (85.6)	30 233 (86.1)	79 155 (87.2)		208 927 (90.9)	40 213 (89.7)	43 663 (90)	125 051 (91.6)
1	16 924 (10.7)	3714 (11.4)	3858 (11)	9352 (10.3)		31 337 (13.6)	5935 (13.2)	8510 (17.5)	16 892 (12.4)
≥2	4219 (2.7)	1006 (2.9)	987 (3)	2226 (2.5)		15 465 (6.7)	3395 (7.6)	3526 (7.3)	8544 (6.3)
Immigration status*:									
UK national	152 637 (96.3)	34 955 (99.6)	32 550 (99.8)	85 132 (93.8)		-	-	-	-
Non-UK national	804 (0.5)	142 (0.4)	65 (0.2)	597 (0.7)		-	-	-	-
Whole or part time*:						-	-	-	-
Whole time	88 634 (55.9)	17 232 (49.1)	20 221 (62)	51 181 (56.4)		-	-	-	-
Part time	64 807 (40.9)	17 865 (50.9)	12 394 (38)	34 548 (38.1)		-	-	-	-

SIMD=Scottish Index of Multiple Deprivation.

* Immigration status and whole or part time status are not included in General Practitioner Contractor Database, so percentages do not sum to 100% for these variables.

We estimated the total Scottish population to be 5 463 300, with the working age population (18-65 years) estimated at 3 452 592 (supplementary figure B). Across the entire Scottish population, 6346 hospital admissions with covid-19 occurred (table 2 and supplementary figure B). REACT-COVID-19 included clinical data on all these cases and 10 randomly selected controls for each case (supplementary figure B).^10^ Of 6346 hospital admissions with covid-19 in Scotland, 33% (n=2097) occurred in the working age population (18-65 years). Of these, 1737 (82.8%) occurred in the general population, and healthcare workers and their household members accounted for 243 (11.6%) and 117 (5.6%) respectively (table 2 and table 3). This meant that healthcare workers and their household members accounted for 17.2% (360/2097) of admissions with covid-19 while representing only 11.2% (388 350/3 452 592) of the working age population. Among household members, a further 24 hospital admissions occurred in 89 327 people below the age of 18 or above 65 years (table 3).

**Table 2:** Association between role and risk of admission to hospital with covid-19 among healthcare workers, members of their households, and the population of Scotland. Values are estimates (95% CIs) unless stated otherwise

Descriptive statistics/model descriptions	Healthcare worker comparison					Household member of healthcare worker comparison			
	General population: other working age residents of Scotland	Non-patient facing	Undetermined	Patient facing		General population: other residents of Scotland (all ages)	Household of non-patient facing healthcare workers	Household of undetermined healthcare workers	Household of patient facing healthcare workers
No admitted to hospital	1737	23	39	181		5962	20	32	89
Total population	3 153 569	32 615	35 097	90 733		5 074 950	44 812	48 530	136 563
Risk of hospital admission (%)	0.06	0.07	0.11	0.20		0.12	0.04	0.07	0.07
Model 1: age and sex	0.92 (0.59 to 1.42)	1	1.64 (0.99 to 2.72)	3.31 (2.13 to 5.13)		0.87 (0.50 to 1.49)	1	1.68 (0.95 to 2.95)	1.82 (1.12 to 2.96)
Model 2: as model 1, plus socioeconomic deprivation and ethnicity	0.90 (0.58 to 1.40)	1	1.54 (0.93 to 2.57)	3.29 (2.12 to 5.10)		0.83 (0.48 to 1.43)	1	1.63 (0.93 to 2.87)	1.80 (1.11 to 2.93)
Model 3: as model 2, plus comorbidity	0.81 (0.52 to 1.26)	1	1.55 (0.93 to 2.59)	3.30 (2.13 to 5.13)		0.86 (0.49 to 1.51)	1	1.60 (0.91 to 2.82)	1.79 (1.10 to 2.91)
Model 4: as model 3, plus occupation	-	1	1.53 (0.91 to 2.56)	3.00 (1.68 to 5.36)		-	1	1.13 (0.36 to 3.53)	1.60 (0.53 to 4.82)
Model 5: as model 4, plus part time status	-	1	1.60 (0.96 to 2.69)	3.06 (1.73 to 5.43)		-	1	1.19 (0.39 to 3.66)	1.60 (0.53 to 4.76)

Estimates and 95% CIs were obtained from Cox models for within healthcare worker and household member comparisons and from conditional logistic regression models for population versus non-patient facing comparison. Conditional logistic regression models were fitted within an incidence density case-control study. These estimates can be interpreted as hazard ratios. Cox models were fitted on entire cohort of healthcare workers and household members respectively. Robust standard errors were generated in Cox models to allow for correlations due to multi-person households. Population denominators were obtained by subtracting 2019 mid-year population estimates from number of healthcare workers and their household members.

**Table 3:** Characteristics of patients admitted to hospital with covid-19 among healthcare workers, members of their households, and working age population of Scotland. Values are numbers (percentages) unless stated otherwise

Characteristics	Population (working age) (n=1737)	Healthcare workers* (n=243)	Household members of healthcare worker* (n=141)
Mean (SD) age, years	52.5 (10.5)	49.2 (10.1)	53.9 (15.0)
Age strata:			
<18 years	-	-	5 (3.5)
18-65 years	1737 (100.0)	243 (100.0)	117 (83.9)
>65 years	-	-	19 (13.5)
Male sex	953 (54.9)	75 (30.9)	113 (80.1)
Comorbidity:			
Ischaemic heart disease	44 (2.5)	8 (3.3)	6 (4.3)
Other heart disease	12 (0.7)	<5	<5
Other circulatory system diseases	3 (0.2)	0	0
Asthma and chronic lower respiratory disease	7 (0.4)	<5	0
Neurological disorders	4 (0.2)	0	0
Malignant neoplasms	6 (0.3)	0	0
Disorders of oesophagus, stomach, and duodenum	1 (0.1)	0	0
Diabetes, type 1	8 (0.5)	<5	<5
Diabetes, type 2	150 (8.6)	15 (6.2)	18 (12.8)
Diabetes, type unknown	5 (0.3)	<5	<5
Any comorbidity	219 (12.6)	28 (11.5)	27 (19.1)
Critical care admission or death:			
Intensive care	279 (16.1)	30 (12.3)	28 (19.9)
Died	227 (13.1)	6 (2.5)	18 (12.8)

* Cells with count less than 5 appear as <5 in accordance with disclosure guidance.

### Risk of hospital admission with covid-19 in healthcare workers

**Fig 1:**
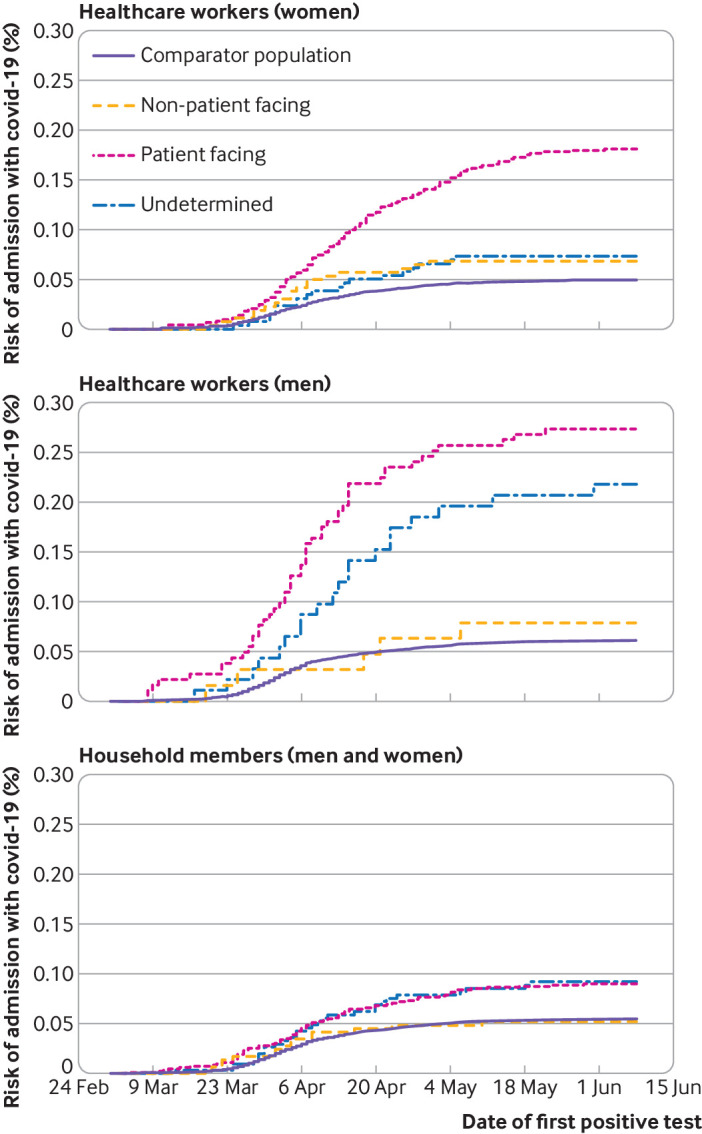
Cumulative incidence (risk) of admission to hospital with covid-19 in healthcare workers, household members of healthcare workers, and the general working age population

The risk of admission to hospital with covid-19 was 0.20% (181/90 733), 0.07% (23/32 615), and 0.11% (39/35 097) in patient facing, non-patient facing, and undetermined healthcare workers (fig 1). With the number of covid-19 infections as the denominator, the risk of hospital admission with covid-19 was 11.5% (23/200) in non-patient facing and 7.3% (181/2485) in patient facing healthcare workers. The rate was 10.5% (39/371) in healthcare workers classified as “undetermined.”

**Fig 2:**
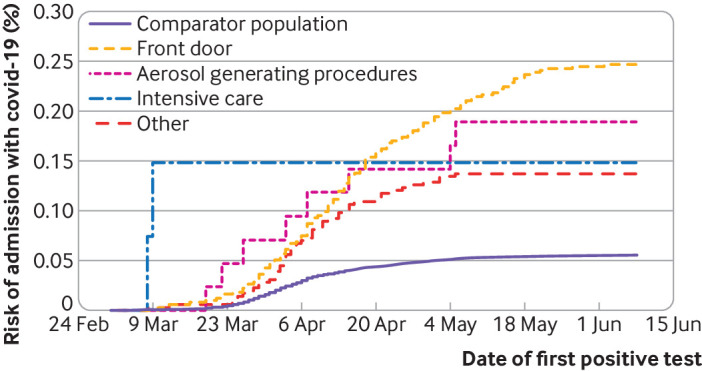
Cumulative incidence (risk) of admission to hospital with covid-19 in patient facing healthcare workers by specific role

Compared with non-patient facing healthcare workers, after adjustment for age, sex, socioeconomic status, ethnicity, and comorbidity, patient facing workers were at a higher risk of hospital admission (hazard ratio 3.30, 95% confidence interval 2.13 to 5.13) (table 2; supplementary table B). We found no evidence of interaction (on the relative scale) by age, sex, or comorbidity (P values 0.57, 0.15, and 0.55, respectively). After adjustment for age, sex, socioeconomic deprivation, and comorbidity, within healthcare workers in patient facing roles, compared with those in the “other” category, front door workers were more likely to be admitted (hazard ratio 2.09, 1.49 to 2.94). For workers in (non-intensive care) aerosol generating procedures roles, the risk was similarly increased, although the confidence interval included the null (hazard ratio 1.91, 0.90 to 4.07). Only 1348 healthcare workers were assigned to the intensive care category, among whom fewer than five hospital admissions occurred, all at an early stage of the pandemic (hazard 1.22, 0.29 to 5.09) (fig 2).

Compared with the general population, the risk among non-patient facing healthcare workers was not increased, including after adjustment for age, sex, socioeconomic deprivation, and comorbidity (hazard ratio 0.81, 0.52 to 1.26) (table 2). Healthcare workers with an undetermined role had an intermediate level of risk between that of patient facing and non-patient facing healthcare workers.

In the cumulative incidence plots (fig 1), the risk seemed to plateau earlier in non-patient facing healthcare workers and in the general population than in patient facing healthcare workers. In exploratory analyses, we therefore compared the risk in patient facing healthcare workers with that in the general population (too few cases occurred in May for models comparing non-patient facing healthcare workers to converge) over time; conditioning on age and sex, the hazard ratios were 2.64 (1.82 to 3.82), 4.18 (3.29 to 5.30), and 6.44 (4.00 to 10.37) for March, April, and May respectively (P for interaction=0.01).

In further exploratory analysis, we evaluated the risk of hospital admission with covid-19 within occupational roles in healthcare workers (using nursing and midwifery staff as the referent). Absolute risk across occupational roles ranged from 0.07% in administrative staff to 0.20% in nursing and midwifery staff (supplementary table C). Given the small number of admissions within some occupational groups, drawing strong conclusions as to whether any specific occupational role carried a higher adjusted risk of admission was difficult. The confidence intervals were wide, and all risk estimates crossed the null.

### Risk of hospital admission with covid-19 in household members of healthcare workers

The risk of admission to hospital with covid-19 was 0.07% (89/136 563), 0.04% (20/44 812), and 0.07% (32/48 530) in household members of patient facing, non-patient facing, and undetermined healthcare workers (fig 1). The overall absolute risk in household members of healthcare workers below the age of 18 years was low (5/78 253; 0.01%).

Associations seen among household members were similar, albeit attenuated, to those seen among healthcare workers. In models adjusting for age and sex, compared with household members of non-patient facing healthcare workers, those in households of patient facing healthcare workers had a higher risk of hospital admission (hazard ratio 1.82, 1.12 to 2.96). We also saw this association after adjusting for age, sex, ethnicity, socioeconomic deprivation, and comorbidity (hazard ratio 1.79, 1.10 to 2.91). Those in households of non-patient facing healthcare workers had a similar risk to that seen in the general population (hazard ratio 0.86, 0.49 to 1.51) (table 2; supplementary table D).

### Age, sex, and comorbidity

**Fig 3:**
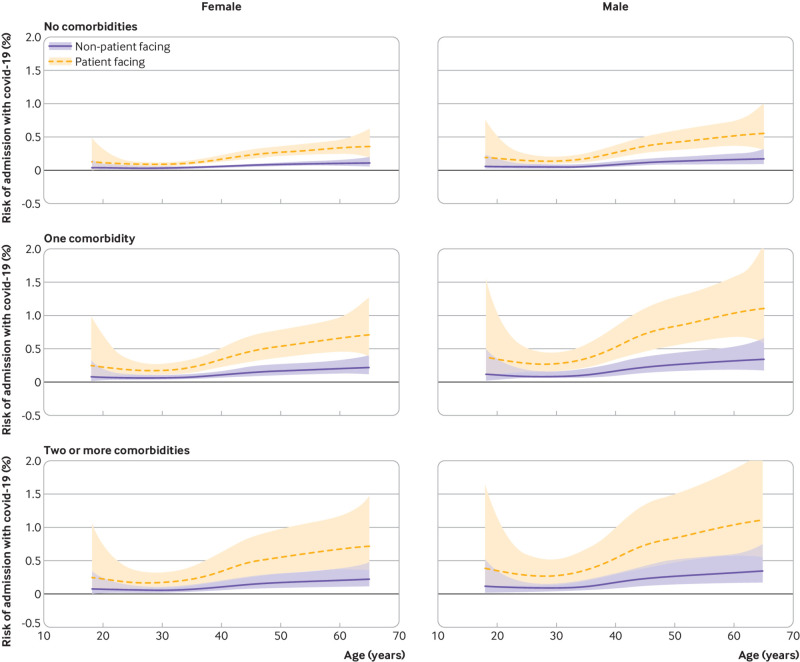
90 day risk of admission to hospital with covid-19 from 1 March 2020 by age, sex, comorbidity count (none, one, or two or more), and occupational role in healthcare workers. Central estimates and 95% CIs were obtained from Cox regression models on age (with penalised splines to allow for non-linearity), sex, and comorbidity count

**Fig 4:**
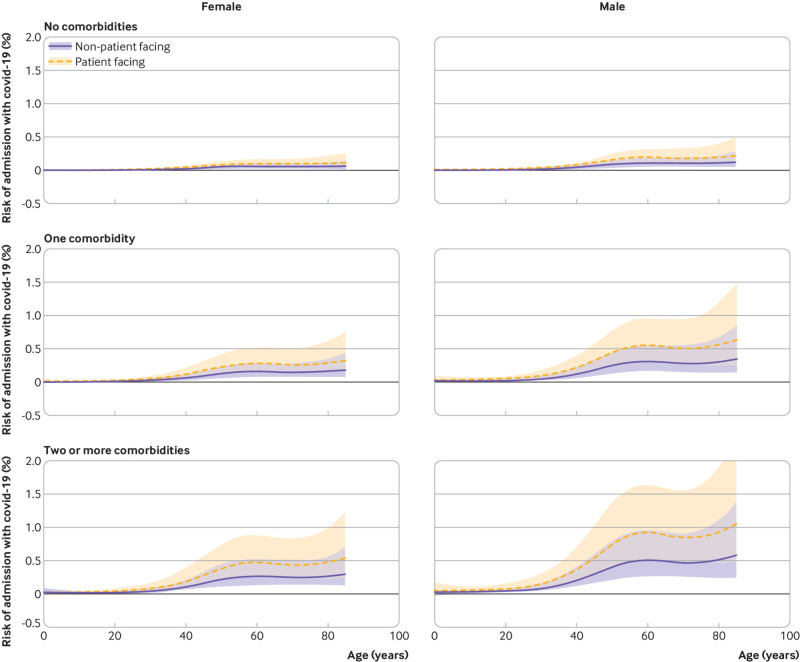
90 day risk of admission to hospital with covid-19 from 1 March 2020 by age, sex comorbidity count (none, one, or two or more), and occupational role in household members of healthcare workers. Central estimates and 95% CIs were obtained from Cox regression models on age (with penalised splines to allow for non-linearity), sex, and comorbidity count

Figure 3 and figure 4 illustrate the absolute 90 day risk (from 1 March 2020) to healthcare workers and their household members based on Cox models adjusting for role, age, sex, and comorbidity. For most healthcare workers and household members, the risks remained below 0.5%. Only older men with at least one comorbidity who were in patient facing roles, or who were household members of a patient facing healthcare worker, had risks approaching 1% or higher. Among patient facing healthcare workers, 5% (4614/90 733) had a household member, or were themselves, in this higher risk group (male, aged 60 years, with one or more comorbidity).

### Characteristics and outcomes of healthcare workers, household members, and general population members admitted to hospital with covid-19

Among hospital admissions with covid-19, compared with the general population, healthcare workers were similar in terms of age and comorbidity (table 3). However, the rates of admission to intensive care were lower (30 (12.3%) in healthcare workers and 279 (16.1%) in the working age population), and a lower proportion of deaths occurred within 28 days (6 (2.5%) *v* 227 (13.1%)). Household members were more similar to the general population.

## Discussion

In nearly 160 000 healthcare workers and 250 000 household members of healthcare workers, we found that admission to hospital with covid-19 was uncommon, with an overall risk of less than 0.5% during the covid-19 pandemic period (1 March 2020 to 6 June 2020). Compared with other adults of working age, however, this risk was higher. Accounting for age, sex, and other confounders, patient facing healthcare workers and members of their households were, respectively, threefold and twofold more likely to be admitted to hospital. Healthcare workers and their households accounted for one in six of all admissions with covid-19 in the working age population (18-65 years).

Across both the general and the healthcare worker populations, the absolute risk of hospital admissions remains relatively small, ranging from 0.06% to 0.20%. This low absolute risk reflects the fact that risk from covid-19 is strongly related to age and that most adults (and all healthcare workers) included our analyses were aged between 18 and 65 years. Nevertheless, within healthcare workers who were admitted to hospital, a non-trivial proportion resulted in admission to critical care or death. Among admitted healthcare workers, one in eight were admitted into critical care and six (2.5%) died; in admitted household members, one in five were admitted to critical care and 18 (12.9%) died. Therefore, as well as having implications for the transmission of covid-19,^3^ ^13^ and the sustainability and deliverability of healthcare,^4^ these findings have implications for the safety and wellbeing of healthcare workers and their households.^14^

### Comparison with other studies and policy implications

We report the risk of covid-19 in nearly 250 000 household members of healthcare workers. Previous evidence on the risk of covid-19 to household members of healthcare workers is sparse,^15^ despite evidence that their safety is of major importance to healthcare workers.^14^ We show that the risk of hospital admission with covid-19 was nearly twofold higher in household members of patient facing compared with non-patient facing healthcare workers. Therefore, the susceptibility of household members, as well as healthcare workers themselves, needs to be considered when assessing occupational risk.

Several studies have reported an increased risk of covid-19 infection and high prevalence of SARS-CoV-2 in healthcare workers, especially in front line workers.^2^ ^5^ ^15^ ^16^ ^17^ ^18^ However, many of these reports were small, single centre, and cross sectional in nature and used methods highly susceptible to bias or restricted their populations to physicians and nurses.^2^ ^5^ ^19^ ^20^ In a large healthcare worker population including a wide range of occupations with robust adjustment for confounding factors, we provide strong evidence that patient facing healthcare workers are at moderately increased risk of experiencing a sufficiently severe form of covid-19 to need hospital admission. We provide further evidence that within patient facing healthcare workers, those categorised as working in “front door” specialties are at the highest risk of admission with covid-19, probably reflecting the higher seroprevalence rates of SARS-CoV-2 in this population.^21^

In response to emerging evidence and international guidance, the NHS in Scotland introduced several changes to infection prevention and control guidance during the course of the pandemic.^22^ Despite this, the differential in risk between the general working age population (who had at this time minimal contacts outside their own households) and patient facing healthcare workers did not fall and may have increased. In contrast, the risk seemed to fall quickly in the “higher risk” intensive care settings. Consistent with international guidance, the NHS in Scotland recommends higher levels of personal protective equipment in higher risk settings, such as intensive care.^22^ In this context, it is notable that less than five healthcare workers based in intensive care were admitted to hospital, all of whom first tested positive for SARS-CoV-2 in early March. In view of the small numbers of staff in intensive care settings, considerable caution is needed in interpreting this finding, but it is consistent with a recent report from Wuhan that no healthcare workers in high risk clinical areas tested positive for SARS-CoV-2 in the context of robust infection control measures being in place.^23^ Together with the observations that the relative risk, compared with the general population, in patient facing healthcare workers continued to rise during the course of pandemic and that the overall risk was highest in front door healthcare workers, these findings raise particular concerns about moderate exposure settings, in terms of both the risk to staff and the risk of transmitting infection to the wider community.

In moderate risk settings, where patients may have only suspected, or even unsuspected, covid-19, the use of more resource intensive and burdensome personal protective equipment of the kind deployed in high risk settings is very challenging.^24^ ^25^ One proposed alternative, or additional, measure to improve safety is therefore to redeploy healthcare workers from patient facing to non-patient facing roles if they or their households are more susceptible to severe disease. Our findings suggest that this may be a feasible policy for two reasons. Firstly, non-patient facing healthcare workers and their households had similar risks of hospital admission to the general population. Secondly, the proportion of patient facing healthcare workers who themselves, or whose households, were at increased risk of admission (up to 1%) was low at around one in 20.

### Limitations of study

Several limitations need to be considered. Firstly, given the small number of deaths in the healthcare worker population, we were unable to estimate the risk of covid-19 related mortality compared with the general population. The Office for National Statistics (ONS) in England did not find increased covid-19 mortality among healthcare workers.^26^ Several reasons exist why hospital admission might be increased without an increase in deaths. Although we identified a cohort of healthcare workers, and sub-divided these by occupational roles, finding a risk only in patient facing healthcare workers, the ONS study relied on self-reporting for the population at risk, with information provided by the next of kin at registration. The ONS also reported mortality for healthcare workers regardless of their role.^26^ Furthermore, healthcare workers may present earlier, improving their survival for a given severity of covid-19, and/or they may have a lower threshold for admission. Secondly, we defined cases in our cohort on the basis of positive tests for SARS-CoV-2. The sensitivity of polymerase chain reaction tests for SARS-CoV-2 is 80-90% depending on the testing strategy,^27^ meaning that a proportion of true cases would have been misclassified. Thirdly, although we saw clear differences in risks across different exposure groups (patient facing and non-patient facing), and even within patient facing groups (for example, front door versus others), individuals within these groups will have differed in terms of the amount of time they spent in close contact with patients with covid-19. Our datasets were unable to define this degree of exposure. Therefore, in applying our findings, health service providers should consider how typical a healthcare worker is with respect to other healthcare workers in meeting our exposure definitions. Fourthly, given that the healthcare workers in our cohort were predominantly white, our analysis lacked power to comment on the risk of hospital admission in ethnic minority groups.^28^ Finally, we were unable to identify healthcare workers who would have been redeployed or advised to shield. Not accounting for this measure would have likely attenuated our risk estimates.

### Conclusions

As the northern hemisphere enters winter and non-pharmacological measures in populations are relaxed, governments, healthcare managers, and occupational health specialists need to consider how best to protect healthcare workers in the event of a resurgent pandemic. This is necessary to protect the healthcare workers and their families,^14^ in addition to reducing onward transmission into the community,^4^ ^13^ and to maintain a functioning healthcare system. Our findings from the “first wave” in Scotland show that healthcare workers in patient facing roles—especially those in “front door” roles—are, along with their households, at particular risk. Crucially, those in non-patient facing roles had similar risks to the general population. These findings should inform decisions about the organisation of health services, the use of personal protective equipment, and redeployment.

## What is already known on this topic

Several systematic reviews and reports have summarised studies of covid-19 infections in healthcare workers
Most studies have been small, based in single centres, and cross sectional in nature and used methods highly susceptible to bias or restricted their populations to physicians and nurses
Studies evaluating the risk of covid-19 infection in household members of healthcare workers are lacking

## What this study adds

Healthcare workers and their households contributed a sixth of hospital admissions with covid-19 among working age adults
Healthcare workers in patient facing roles—especially those in “front door” roles—are, along with their households, at higher risk of admission with covid-19
Importantly, those in non-patient facing roles had similar risks to the general population

## Web extra

Extra material supplied by authors

Web appendix: Supplementary appendices, tables, and figures

Web appendix: Statistical analysis plan

## Notes

Contributors: DAM conceived and ASVS, RW, CF, CR, SH, PM, HC, and DAM designed the study. ASVS, DAM, CG, DC, JB, AW, SK and MR analysed and interpreted the data. DAM and ASP drafted the first version of the manuscript. All of the authors contributed to, read, and approved the final manuscript. The corresponding author attests that all listed authors meet authorship criteria and that no others meeting the criteria have been omitted. DAM is the guarantor.

Ethical approval: This project was approved by the Public Benefit and Privacy Panel (2021-0013)

Data sharing: The analysis code is available at https://github.com/ChronicDiseaseEpi/hcw. As the analysis involved data on unconsented participants, individual level data cannot be shared.

The corresponding author affirms that this manuscript is an honest, accurate, and transparent account of the study being reported; that no important aspects of the study have been omitted; and that any discrepancies from the study as planned (and, if relevant, registered) have been explained.

Dissemination to participants and related patient and public communities: The study results will be shared with the professional bodies relevant to the research participants.

Provenance and peer review: Not commissioned; externally peer reviewed.
