# Supplementary material for: Risk of hospital admission with coronavirus disease 2019 in healthcare workers and their households: nationwide linkage cohort study
Source: BMJ. 2020 Oct 28;371:m3582. doi: 10.1136/bmj.m3582 (PMC7591828; doi:10.1136/bmj.m3582)
Supplement: Supplementary file 1 — Web appendix: Supplementary appendices, tables, and figures [file shaa060836.ww1.pdf]

SUPPLEMENTARY MATERIAL

Risk of hospitalization with coronavirus disease 2019 in healthcare workers and their household members: a nationwide linkage cohort study

Shah ASV[1,2], Wood R[3,4], Gribben C[3], Caldwell D[3], Bishop J[3], Weir A[3], Kennedy S[3], Martin R[3], Smith-Palmer A[3], Goldberg D[3], McMenamin J[3], Fischbacher C[3], Robertson C[3], Hutchinson S[3,5], McKeigue P[6], Colhoun H[3,7], McAllister DA[3,8]

1. Non-communicable Disease Epidemiology, London School of Hygiene and Tropical Medicine, London, UK.
2. Department of cardiology, Imperial College NHS Trust
3. Public Health Scotland, Edinburgh, UK.
4. Centre for Population Health Sciences, University of Edinburgh
5. School of Health and Life Sciences, Glasgow Caledonian University, Glasgow, UK.
6. MRC Institute of Genetics & Molecular Medicine, University of Edinburgh, Edinburgh, UK.
7. Usher Institute, University of Edinburgh, Edinburgh, UK.
8. Institute of Health and Wellbeing, University of Glasgow, Glasgow, UK.

Correspondence and requests for reprints:
Dr David McAllister
Institute of health and well being
University of Glasgow,
Glasgow
United Kingdom
E-mail: david.mcallister@glasgow.ac.uk

All analyses code is available at our github repository.

Appendix 1

Descriptions and sources of national databases used for individual patient level linkage

<u>Scottish hospitalization record from SMR01</u>

Comorbidities were defined using the Scottish morbidity record 01 (SMR01) - General/Acute Inpatient & Day Case. SMR01 an episode-based patient record relating to all inpatients and day cases discharged from non-obstetric and non-psychiatric specialties. A record is generated when a patient completes an episode of inpatient or day case care. Data collected include patient identifiable and demographic details, episode management details and general clinical information. Currently diagnoses are recorded using the ICD-10 classification and operations are recorded using the OPCS-4 classification. Further information on the national dataset and variables contained is available at https://www.ndc.scot.nhs.uk/Data-Dictionary/SMR-Datasets//Episode-Management/SMR-Record-Type/

<u>National Records of Scotland (NRS)</u>

The NRS covers all deaths in Scotland with approximately 55,000 deaths registered annually. The National Records of Scotland Death Records are linked with the NHS Scotland Scottish Morbidity Database which links together NHS Scotland inpatient, mental health and cancer registry datasets with the NRS Death Records. NRS records death status, cause of death and date of death.

Further information of the NRS death registry is available at https://www.ndc.scot.nhs.uk/National-Datasets/data.asp?SubID=13

<u>Prescribing Information System (PIS)</u>

The Prescribing Information System (PIS) is the definitive data source for all prescribing relating to all medicines and their costs that are prescribed and dispensed in the community in Scotland. The information is supplied by Practitioner & Counter Fraud Services Division (P&CFS) who is responsible for the processing and pricing of all prescriptions dispensed in Scotland. Primary care physicians write the vast majority of these prescriptions, with the remainder written by other authorised prescribers such as nurses and dentists. Also included in the dataset are prescriptions written in hospitals that are dispensed in the community. Note that prescriptions dispensed within hospitals are not included.

Further information on the Prescribing Information System operational in Scotland is available at https://www.ndc.scot.nhs.uk/National-Datasets/data.asp?SubID=9

<u>National microbiology register (Electronic Communication of Surveillance in Scotland [ECOSS])</u>

The Scottish microbiology surveillance registry, or '*Electronic Communication of Surveillance in Scotland*' (ECOSS) as it is termed by NHS National Services Scotland, was used in the present study to provide individual patient-level data on SARS-Cov-2 testing and results. ECOSS is part of NHS Scotland's Infection Intelligence Platform (IIP),[1,2] which was

set-up in response to the UK's antimicrobial resistance (AMR) strategy (2013-2018) with the aim of providing "*better access to and use of surveillance data*".[3]

Data were first collected and recorded within ECOSS in 2007. The dataset is maintained by NHS National Services Scotland on behalf of Public Health Scotland (formerly Health Protection Scotland). ECOSS is updated monthly and, as of 2017, it contained approximately 29 million records of positive microbiology laboratory specimens from across Scotland.[1] It provides data for numerous national clinical and research activities, audit projects and Scottish Government reports, including: the identification of cases of severe infectious disease, infectious disease outbreaks and the evaluation of longer term trends in the incidence of laboratory-reported infections; surveillance of episodes of Clostridium difficile infections, Escherichia coli bacteraemia, Staphylococcus aureus bacteraemia and surgical site infections.[2] NHS National Services Scotland monitors the completeness and accuracy of ECOSS data through its 'Data Monitoring and Support Service'.[1] Further, NHS National Services Scotland routinely informs data users of any problems affecting the accuracy or assurance of these data.

More information on the ECOSS data system is available at https://www.hps.scot.nhs.uk/data/

Scottish Workforce Information Standard System (SWISS)

The Scottish Workforce Information Standard System (SWISS) is a national human resources database held by NHS Education Scotland which contains data on all directly employed staff (ie not contracted staff such as general practitioners except where they are also directly employed in some other role) working in the NHS in Scotland. It includes data for territorial health boards, and boards providing a national service. It records the job title using a nationally agreed standard as well as, for medical and dental staff the medical specialty. It also includes data on occupation grade, part-time/whole time status, and the designated service area.

More information on the SWISS database is available at https://turasdata.nes.nhs.scot/media/2prjxbg4/2020-06-02-workforce-report.pdf.

General Practitioner Contractor Database (GPCD)

The General Practitioner Contractor Database (GPCD) includes all contracted general practitioners working in Scotland. This includes GP partners who are independent contractors, and salaried GPs, but not locum GPs. GOs in training grades are employed centrally and so are included in SWISS.

More information on the GPCD database is available at https://www.isdscotland.org/Health-Topics/General-Practice/Workforce-and-Practice-Populations/.

Rapid preliminary inpatient data (RAPID)

The RAPID database has been operational since to 2001 to monitor and predict emergency admissions and bed occupancy across National Health Service Boards in Scotland. Data from this database has already been used to provide information to NHS boards, healthcare workers and the public on the direct and indirect effects of the COVID-19 pandemic. These

dashboards can be found at: https://publichealthscotland.scot/our-areas-of-work/sharing-our-data-and-intelligence/coronavirus-covid-19-data/

In 2015 the data collection was expanded to include data items on individual patient level data on age, sex, times of admission and discharge, ward significant facility, diagnosis and operation codes and information on patient discharge.

Further information on the RAPID databases can be found here:
https://www.ndc.scot.nhs.uk/National-Datasets/data.asp?ID=1&SubID=37

References

1. Bennie M, Malcolm W, Marwick CA, Kavanagh K, Sneddon J, Nathwani D. Building a national Infection Intelligence Platform to improve antimicrobial stewardship and drive better patient outcomes: the Scottish experience. *J Antimicrob Chemother*. 2017 Oct 1;72(10):2938-42.
2. NHS National Services Scotland (NSS), 2014. Infection Intelligence Platform (IIP)- High level guide to IIP component datasets held by NHS National Services Scotland, https://www.isdscotland.org/Health-Topics/Health-and-Social-Community-Care/Infection-Intelligence-Platform/Data/June-2014-Guide-to-IIP-Data.pdf (accessed 18th March, 2020)
3. Scottish Government, 2014. Scottish Management of Antimicrobial Resistance Action Plan 2014-2018. http://www.gov.scot/Publications/2014/07/9192 (accessed 2nd March, 2020)

Appendix 2

The following tables show the exclusions from the Scottish Workforce Information Standard System (SWISS) and General Practitioner Contractor Database (GPCD), and their linkage to the Community Health Index (CHI) database. Note that pediatric staff were excluded where they *exclusively* worked in pediatrics.

.

Medical staff on SWISS database

Selection of medical and dental from SWISS	16,499
Drop staff where the grades clash	16,433
Drop implausible specialty combinations	16,402
Drop medical directors	16,379
Drop GPs in non-training grades	15,807
Drop where there is no specialty data	14,421
Final medical dataset	14,421

Non-medical staff on SWISS database

Non-medical	150,131

Combined staff on SWISS database

Non-medical and medical	164,552
Not in both medical and non-medical (one person)	164,550
Match in CHI database	162,945
Match in CHI database non-medical	148,930
Match in CHI database medical	14,015

General Practice Contractor Database

General practice doctors	5,043
Not in medical or non-medical SWISS database	4,378
Of above, those which are household members of medical or non-medical	596

GPCD and SWISS

General practice doctors and medical and non-medical (162945 + 4378)	167,323
Exclude dental and paediatric*	160,971
Exclude aged <18 or >65	158,445

**** Dental healthcare workers were excluded from all analyses because only a small proportion of the dental workforce is captured in the SWISS database and the majority of dental services were closed during the COVID-19 pandemic. Paediatric healthcare workers were excluded because children differ from adults in their propensity to transmit COVID-19 infection and, compared to the 160,971 NHS staff in Scotland, there were comparatively few staff solely working in paediatric roles.***

Appendix 3: Definitions used to categorize healthcare workers and list of specialties, job roles and service areas by categorization

These tables are also available in machine readable formats in our public-facing github repository (https://github.com/ChronicDiseaseEpi/hcw).

Definition of categorization for patient and non-patient facing and undetermined healthcare workers

Definition	Description	Definition
Any staff	Any member of NHS staff.	Included in SWISS or GPCD database
Patient facing, any	Include if likely to currently be working in patient-facing role.	Specific list of AFC roles and/or medical specialties (see below)
Non-patient-facing	Any member of NHS staff likely to be in a non-patient-facing role.	Specific list of AFC roles and/or medical specialties (see below)
Undetermined	Staff where it is not possible to allocate with confidence to patient-facing or non-patient-facing roles	Any staff not in “Patient-facing, any” or “Non-patient-facing” (see below)

Definition of categorization for patient facing roles into those at front door, exposed to aerosolized generating procedures (AGP), in intensive care and other

Definition	Description	Definition*	Comparator(s)
Patient-facing, front-door COVID19	Include if involved in acute medical receiving of patients with possible or probable COVID19 (ie not incidental finding such as COVID19 in patient with myocardial infarction).	Specific list of AFC roles and/or medical specialties and/or designated service area (see below).	Patient-facing, other
Patient-facing, Intensive care	Intensive care medicine and anaesthetic specialties	Specific list of AFC roles and/or medical specialties and/or designated service area (see below).	Patient-facing, other
Patient-facing, Resp-oral-nasal-AGP	Include if involved in work with high risk of exposure to oral, nasal or respiratory secretions and/or aerosol generating procedures (AGP) outwith intensive care settings.	Specific list of AFC roles and/or medical specialties and/or designated service area (see below).	Patient-facing, other
Patient-facing, other	Patient-facing role but not front-door COVID or resp-oral-nasal-AGP or intensive care.	Patient-facing role but not front-door COVID or resp-oral-nasal-AGP or intensive care.	

*Nursing staff in the General Acute Nursing and Specialist Nursing and have been further assigned to specific roles according to the recorded service area. This was done for the following territorial Health Boards, in whom there is >= 95% completeness for the service area variable: - NHS Ayrshire & Arran, NHS Borders, NHS Dumfries & Galloway, NHS Forth Valley, NHS Grampian, NHS Greater Glasgow & Clyde, NHS Highland, NHS Orkney and NHS Shetland. The remaining territorial boards had lower completeness for service area (ranging from 91% to <1%)

*Definition of medical specialties into patient facing and non-patient facing roles**

	Non patient-facing	Patient-facing**			
		Any	Front-door	Resp-oral-nasal-AGP	Intensive care
Acute Internal Medicine		Yes	Yes		
Allergy					
Anaesthetics		Yes			Yes
Audio Vestibular Medicine					
Audiological Medicine					
Blood Transfusion					
Breast Screening Service					
Cardiology		Yes			
Cardiothoracic Surgery		Yes			
Chemical Pathology					
Clinical Genetics					
Clinical Neurophysiology					
Clinical Oncology		Yes			
Clinical Pharmacology and Therapeutics		Yes			
Clinical Radiology					
Community Psychiatry		Yes			
Community Sexual And Reproductive Health		Yes			
Dermatology					
Diagnostic Neuropathology	Yes				
Emergency Medicine		Yes	Yes		
Endocrinology and Diabetes		Yes			
Endodontics		Yes		Yes	
Ent Surgery		Yes		Yes	
Family Planning Service					
Fixed & Removable Prosthodontics					
Forensic Histopathology	Yes				
Forensic Psychiatry					
Gastroenterology		Yes			
General (Internal) Medicine		Yes	Yes		
General Psychiatry		Yes			
General Surgery		Yes			
Genito-Urinary Medicine		Yes			
Geriatric Medicine		Yes	Yes		
GP Other Than Obstetrics		Yes			

Haematology					
Histopathology	Yes				
Homeopathy					
Immunology					
Infectious Diseases		Yes	Yes		
Intensive Care Medicine		Yes			Yes
Medical Microbiology And Virology					
Medical Oncology		Yes			
Medical Ophthalmology		Yes			
Microbiology					
Neurology		Yes			
Neurosurgery		Yes			
Nuclear Medicine					
Obstetrics And Gynaecology		Yes			
Occupational Medicine		Yes			
Old Age Psychiatry		Yes			
Ophthalmology		Yes			
Oral And Maxillofacial Surgery		Yes		Yes	
Oral And Maxillofacial Pathology	Yes				
Oral Medicine		Yes		Yes	
Oral Microbiology					
Oral Pathology					
Oral Surgery		Yes		Yes	
Orthodontics		Yes		Yes	
Otolaryngology		Yes		Yes	
Pain Management					
Palliative Medicine					
Plastic Surgery		Yes			
Psychiatry Of Learning Disability					
Psychotherapy					
Public Health Medicine	Yes				
Rehabilitation Medicine		Yes			
Renal Medicine		Yes			
Respiratory Medicine		Yes		Yes	
Restorative Dentistry					
Rheumatology		Yes			
Special Care Dentistry		Yes		Yes	
Surgical Dentistry		Yes		Yes	
Trauma And Orthopaedic Surgery		Yes			
Urology		Yes			
Vascular Surgery		Yes			
Virology					

There are two specialty fields in the SWISS database. These are “specialty” with around 85% completeness, and “second specialty” with lower completeness. We added data from TURAS

People (which holds data on doctors in training roles) to increase the completeness to approximately 98%.

** Specialities unable to be to categorized into patient facing or non-patient facing roles were categorized into undetermined.*

***Patient facing medical specialties were categorized into front door, specialties exposed to aerosalized generating procedures and intensive care. Remaining specialties were categorized into 'other'*

*Definition of nursing and midwifery, allied health professionals and support services into patient facing and non-patient facing roles**

		Non patient-facing	Patient facing**			
Job Family	Job Sub Family		Any	Front-door	Resp-oral-nasal-AGP	Intensive care
ADMINISTRATIVE SERVICES	FINANCE	Yes				
ADMINISTRATIVE SERVICES	HUMAN RESOURCES	Yes				
ADMINISTRATIVE SERVICES	INFORMATION SYSTEMS/TECHNOLOGY	Yes				
ADMINISTRATIVE SERVICES	NA	Yes				
ADMINISTRATIVE SERVICES	NHS24 CALL HANDLER	Yes				
ADMINISTRATIVE SERVICES	OFFICE SERVICES	Yes				
ADMINISTRATIVE SERVICES	PATIENT SERVICES	Yes				
ALLIED HEALTH PROFESSION	AHP TRAINING/ADMINISTRATION	Yes				
ALLIED HEALTH PROFESSION	AMBULANCE PARAMEDIC		Yes	Yes		
ALLIED HEALTH PROFESSION	ARTS THERAPIES					
ALLIED HEALTH PROFESSION	DIAGNOSTIC RADIOGRAPHY		Yes			
ALLIED HEALTH PROFESSION	DIETETICS					
ALLIED HEALTH PROFESSION	GENERIC THERAPIES					
ALLIED HEALTH PROFESSION	OCCUPATIONAL THERAPY		Yes			
ALLIED HEALTH PROFESSION	ORTHOPTICS					
ALLIED HEALTH PROFESSION	ORTHOTICS		Yes			
ALLIED HEALTH PROFESSION	PHYSIOTHERAPY		Yes		Yes	
ALLIED HEALTH PROFESSION	PODIATRY		Yes			
ALLIED HEALTH PROFESSION	PROSTHETICS					
ALLIED HEALTH PROFESSION	SPEECH AND LANGUAGE THERAPY		Yes			
ALLIED HEALTH PROFESSION	THERAPEUTIC RADIOGRAPHY		Yes	Yes		

AMBULANCE SERVICES	AMBULANCE CARE ASSISTANT		Yes	Yes		
AMBULANCE SERVICES	AMBULANCE TECHNICIAN		Yes	Yes		
AMBULANCE SERVICES	DRIVER		Yes	Yes		
AMBULANCE SERVICES	EMDC OPERATIVE	Yes				
AMBULANCE SERVICES	OPERATIONAL MANAGER	Yes				
AMBULANCE SERVICES	PTS DAY CONTROL	Yes				
EMERGENCY SERVICES	AMBULANCE AUXILIARY		Yes	Yes		
EMERGENCY SERVICES	AMBULANCE CARE ASSISTANT		Yes	Yes		
EMERGENCY SERVICES	AMBULANCE PARAMEDIC		Yes	Yes		
EMERGENCY SERVICES	AMBULANCE TECHNICIAN		Yes	Yes		
EMERGENCY SERVICES	DRIVER		Yes	Yes		
EMERGENCY SERVICES	EMDC OPERATIVE	Yes				
EMERGENCY SERVICES	OPERATIONAL MANAGER	Yes				
EMERGENCY SERVICES	PTS DAY CONTROL					
HEALTHCARE SCIENCES	BIOMEDICAL SCIENCES LIFE					
HEALTHCARE SCIENCES	CLIN PHOTO/ILLUSTRATE PHYSICAL					
HEALTHCARE SCIENCES	CLINICAL PERFUSION PHYSIOLOGY					
HEALTHCARE SCIENCES	CLINICAL PHYSIOLOGY					
HEALTHCARE SCIENCES	CLINICAL SCIENCES LIFE					
HEALTHCARE SCIENCES	CLINICAL SCIENCES PHYSICAL					
HEALTHCARE SCIENCES	CLINICAL SCIENCES PHYSIOLOGY					
HEALTHCARE SCIENCES	CLINICAL TECHNOLOGY LIFE					
HEALTHCARE SCIENCES	CLINICAL TECHNOLOGY PHYSICAL					
HEALTHCARE SCIENCES	MAXILLOFACIAL PROS PHYSICAL					
HEALTHCARE SCIENCES	NA					
HEALTHCARE SCIENCES	STERILE SERVICES LIFE					
MEDICAL AND DENTAL SUPPORT	DENTAL NURSING		Yes		Yes	

MEDICAL AND DENTAL SUPPORT	DENTAL TECHNOLOGY		Yes		Yes	
MEDICAL AND DENTAL SUPPORT	OPERATING DEPARTMENT		Yes			
MEDICAL AND DENTAL SUPPORT	ORAL HEALTH		Yes		Yes	
MEDICAL AND DENTAL SUPPORT	PHYSICIANS ASSISTANT					
MEDICAL AND DENTAL SUPPORT	THEATRE SERVICES		Yes			
MEDICAL SUPPORT	OPERATING DEPARTMENT		Yes			
MEDICAL SUPPORT	PHYSICIANS ASSISTANT		Yes			
MEDICAL SUPPORT	THEATRE SERVICES		Yes			
NURSING AND MIDWIFERY	COMMUNITY CHILDREN'S NURSING					
NURSING AND MIDWIFERY	MIDWIFERY DIRECT CC		Yes			
NURSING AND MIDWIFERY	MIDWIFERY INDIRECT CC		Yes			
NURSING AND MIDWIFERY	NA					
NURSING AND MIDWIFERY	NEONATAL MIDWIFERY CC		Yes			
NURSING AND MIDWIFERY	NEONATAL MIDWIFERY DIRECT CC		Yes			
NURSING AND MIDWIFERY	NEONATAL MIDWIFERY INDIRECT CC		Yes			
NURSING AND MIDWIFERY	NEONATAL NURSING DIRECT CC		Yes			
NURSING AND MIDWIFERY	NEONATAL NURSING INDIRECT CC		Yes			
NURSING AND MIDWIFERY	NHS 24 NURSING	Yes				
NURSING AND MIDWIFERY	NURSING TRAINING/ADMIN/MGT					
NURSING AND MIDWIFERY	PAEDIATRIC NURSING		Yes	Yes		
NURSING AND MIDWIFERY	PRACTICE NURSING		Yes			
NURSING AND MIDWIFERY	PUBLIC HEALTH NURSING					
NURSING AND MIDWIFERY	SCHOOL NURSING					
NURSING AND MIDWIFERY	SEXUAL AND REPRODUCTIVE HEALTH					
NURSING AND MIDWIFERY	SPECIALIST NURSING		Yes			
NURSING AND MIDWIFERY	STAFF NURSERY					
NURSING AND MIDWIFERY	TREATMENT ROOM NURSING		Yes			

NURSING AND MIDWIFERY	BANK NURSING		Yes			
NURSING AND MIDWIFERY	BTS NURSING		Yes	Yes		
NURSING AND MIDWIFERY	CARE OF THE ELDERLY NURSING		Yes	Yes		
NURSING AND MIDWIFERY	COMMUNITY GENERAL NURSING		Yes			
NURSING AND MIDWIFERY	DISTRICT NURSING		Yes			
NURSING AND MIDWIFERY	FAMILY PLANNING NURSING					
NURSING AND MIDWIFERY	GENERAL ACUTE NURSING		Yes	Yes		
NURSING AND MIDWIFERY	HEALTH VISITOR NURSING					
NURSING AND MIDWIFERY	LEARNING DISABILITIES NURSING		Yes			
NURSING AND MIDWIFERY	MENTAL HEALTH NURSING		Yes			
NURSING AND MIDWIFERY	MIDWIFERY		Yes			
OTHER THERAPEUTIC	GENETIC COUNSELLING					
OTHER THERAPEUTIC	NA					
OTHER THERAPEUTIC	OPTOMETRY					
OTHER THERAPEUTIC	PHARMACY					
OTHER THERAPEUTIC	PHARMACY TECHNICIANS	Yes				
OTHER THERAPEUTIC	PLAY SPECIALIST		Yes			
OTHER THERAPEUTIC	PSYCHOLOGY					
PERSONAL AND SOCIAL CARE	CARE AT HOME		Yes			
PERSONAL AND SOCIAL CARE	HEALTH PROMOTION					
PERSONAL AND SOCIAL CARE	HOSPITAL CHAPLAINCY					
PERSONAL AND SOCIAL CARE	RESIDENTIAL / DAY CARE		Yes			
PERSONAL AND SOCIAL CARE	SOCIAL WORK					
SENIOR MANAGERS	NA	Yes				
SUPPORT SERVICES	CATERING SERVICES					
SUPPORT SERVICES	DOMESTIC SERVICES					
SUPPORT SERVICES	ESTATES					

SUPPORT SERVICES	GENERAL SERVICES					
SUPPORT SERVICES	GROUNDS SERVICES					
SUPPORT SERVICES	HOTEL SERVICES					
SUPPORT SERVICES	LAUNDRY/LINEN SERVICES					
SUPPORT SERVICES	NA					
SUPPORT SERVICES	PORTERING SERVICES		Yes			
SUPPORT SERVICES	SECURITY SERVICES					
SUPPORT SERVICES	STERILE SERVICES					
SUPPORT SERVICES	STORES SERVICES					
SUPPORT SERVICES	TRANSPORT SERVICES					
UNALLOCATED / NOT KNOWN	NA					
UNALLOCATED / NOT KNOWN	NOT KNOWN					

** Roles unable to be to categorized into patient facing or non-patient facing were categorized into undetermined.*

***Patient facing roles were categorized into front door, specialties exposed to aerosalized generating procedures and intensive care. Remaining specialties were categorized into 'other'*

Definition of relevant service areas

	Front –door	Respiratory-oro-aerosol – generating procedures	Intensive care
Accident and Emergency	Yes		
Anaesthetics			Yes
Ear Nose & Throat		Yes	
Endocrinology & Diabetes			
Gastroenterology			
General Medicine	Yes		
Infectious Diseases	Yes		
Intensive Care			Yes
Neonatal/SCBU			Yes
Oral & Maxillofacial		Yes	
Respiratory		Yes	
Restorative Dentistry		Yes	

Rheumatology			
Surgical Dentistry		Yes	

Appendix 4: Changes in infection prevention and control guidance in Scotland

The following text was taken from guidance documents produced by Health Protection Scotland and (when this joined with other NHS bodies to form Public Health Scotland) Public Health Scotland.

Version	Date	Staff	Clinicians	Visitors
	23-Jan-20	Ensure that staff are:<br>- Familiar with all Personal Protective Equipment (PPE) required including, provision of adequate supplies, safe donning and removal procedures, where stored and how it should be used;<br>· Aware of what actions to take if a case presents;<br>· Aware of where a case will be isolated and the need for a negative pressure room, if available;<br>· Familiar with FFP3 respirator use and that fit testing and checking has been undertaken before using this equipment	Clinicians must:<br>- Admit patients requiring admission directly to a negative pressure isolation room. If this is not possible then a single room with en-suite facilities should be used. The room door must be kept closed.<br>- Wear appropriate PPE: as a minimum, this should be a FFP3 respirator, disposable, long-sleeved, fluid resistant surgical gown, disposable gloves and eye/face protection.<br>- Ask the patient (if tolerable) to wear a FRSM while being transported to the isolation room.	
5.0	07-Feb-20	Same as above	Same as above	
6.2	02-Mar-20	Same as above	Same as above	
6.6	12-Mar-20	Same as above	Clinicians must:<br>Same as above **plus**<br>- Wear appropriate PPE	If the person is admitted visitors should be restricted to essential visitors only; such as parents of paediatric patients or an affected patient's partner/main carer. Local risk assessment and practical management should be considered, ensuring a pragmatic and proportionate response, including the consideration of whether there is a requirement for visitors to wear PPE including RPE. These visitors must not visit any other care areas or facilities. A log of all visitors should be kept.
7.0	13-Mar-20	Same as above	Same as above	
7.1	14-Mar-20	Same as above	Clinicians must:<br>Same as above **plus**<br>- Assess individuals in a single occupancy room.	
8.0	16-Mar-20	Same as above	Same as above	
8.1	19-Mar-20	Same as above **plus**:<br>Staff who are pregnant or otherwise immunosuppressed should not provide direct care for a patient with possible or confirmed COVID-19, this includes obtaining samples. Any deviation from this should be a local decision. Pregnant staff or staff who are immunosuppressed should seek advice from the local Occupational Health Department.	Same as above	
8.2	27-Mar-20	Same as above plus<br>Staff with underlying health conditions that put them at increased risk of severe illness from COVID-19, including those who are immunosuppressed, should not provide direct care to patients with possible or confirmed COVID-19. Staff who think they may be at	Same as above	

		increased risk should seek advice from their line manager or local Occupational Health service.		
8.3	30-Mar-20		Same as above plus Update on AGP procedures	
9.0	02-Apr-20	Same as above **plus**<br>- COVID-19 Guidance for Infection Prevention and Control in Healthcare Settings **[Item 1]** describes the Infection Prevention and Control measures required for management of possible/confirmed COVID-19 patients.<br>- Table 1 of the guidance **[Item 2]** details the recommended PPE for Healthcare workers within the secondary care inpatient clinical setting within the NHS and independent sectors.<br>Table 4 of the guidance **[Item 3]** provides additional considerations, in addition to standard infection prevention and control precautions, where there is sustained transmission of COVID-19, taking into account individual risk assessment for this new and emerging pathogen, NHS and independent .	Same as above **plus**<br>**Actions to take if possible case definition is met for COVID-19**<br>Ensure the patient is placed in a negative pressure room, a single side room with en-suite facilities or within a specified cohort bay and the PPE described in COVID-19 Guidance for infection prevention and control in healthcare settings **[Item 1]**, is worn by any person entering the room.<br>Ensure that the patient, potentially contaminated areas, and waste are managed as per the infection control guidance.	
9.1	11-Apr-20	Same as above **plus**<br>HCWs who come into contact with a COVID-19 patient or a patient suspected of having COVID-19 while not wearing personal protective equipment (PPE) should follow the advice on the guidance on Management of Exposed Healthcare Workers and Patients in Hospital setting **[Item 4]**	Same as above	
9.2	29-Apr-20	Same as above	Same as above **plus**<br>Staff should be aware of ongoing transmission within the hospital setting. An outbreak is defined as two or more confirmed or suspected cases of COVID-19 where nosocomial infection and ongoing transmission is suspected to have occurred within a 14-day period.	

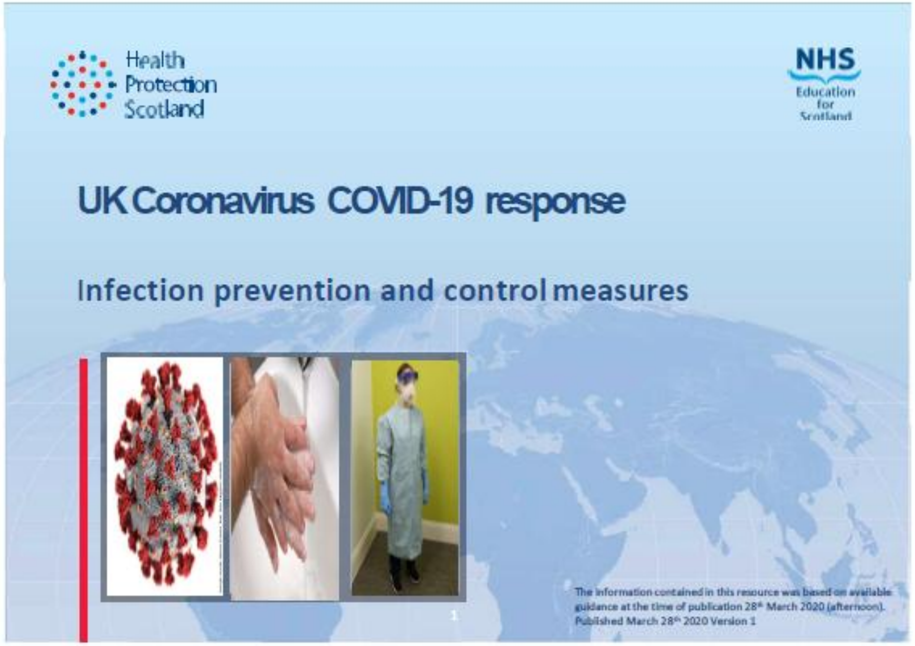

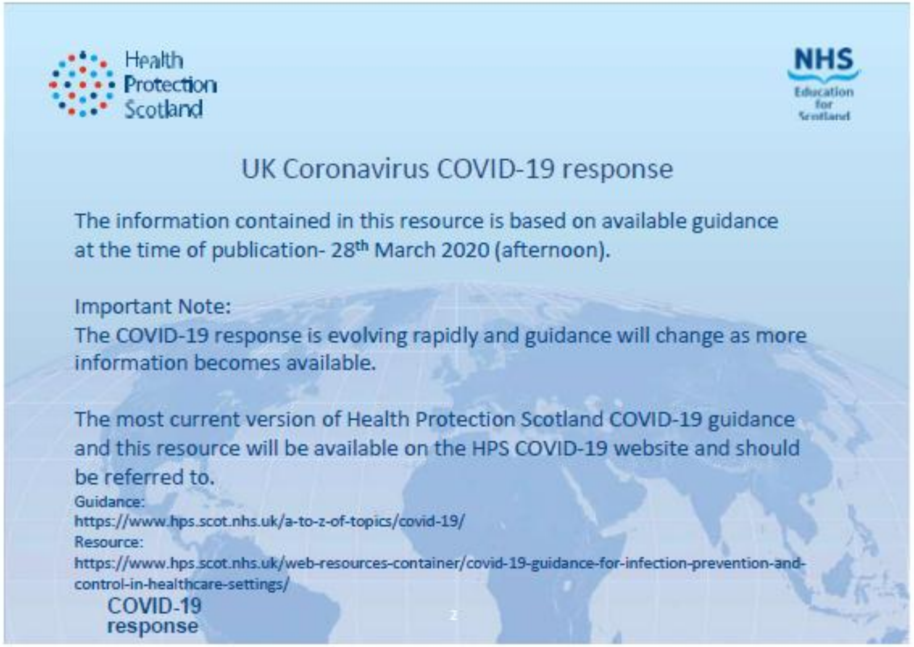

Item 1

The version of the document referred to in the guidance is no longer available at that link (https://www.hps.scot.nhs.uk/web-resources-container/covid-19-guidance-for-infection-prevention-and-control-in-healthcare-settings/), but can obtained on The Internet Archive (https://web.archive.org/) by searching for the url. The first two pages have been reproduced here.

Public Health England | Academy of Medical Royal Colleges | GIG Cymru NHS Wales – Iechyd Cyhoeddus Cymru Public Health Wales | Health Protection Scotland | HSC Public Health Agency | NHS

Recommended PPE for healthcare workers by secondary care inpatient clinical setting, NHS and independent sector

Setting	Context	Disposable Gloves	Disposable Plastic Apron	Disposable fluid-resistant gown	Surgical mask	Fluid-resistant (Type IIR) surgical mask	Filtering face piece respirator	Eye/face protection[1]
Acute hospital inpatient and emergency departments, mental health, learning disability, autism, dental and maternity settings	Performing a single aerosol generating procedure[2] on a possible or confirmed case[3] in any setting outside a higher risk acute care area[4]	✓ single use[5]	✗	✓ single use[5]	✗	✗	✓ single use[5]	✓ single use[5]
	Working in a higher risk acute care area[4] with possible or confirmed case(s)[3]	✓ single use[5]	✓ single use[5]	✓ sessional use[6]	✗	✗	✓ sessional use[6]	✓ sessional use[6]
	Working in an inpatient, maternity, radiology area with possible or confirmed case(s)[3] – direct patient care (within 2 metres)	✓ single use[5]	✓ single use[5]	✗	✗	✓ sessional use[6]	✗	✓ sessional use[6]
	Working in an inpatient area with possible or confirmed case(s)[3] (not within 2 metres)	✗	✗	✗	✗	✓ sessional use[6]	✗	✓ risk assess sessional use[6,7]
	Working in an emergency department/acute assessment area with possible or confirmed case(s)[3] – direct patient care (within 2 metres)	✓ single use[5]	✓ single use[5]	✗	✗	✓ sessional use[6]	✗	✓ sessional use[6]
	All individuals transferring possible or confirmed case(s)[3] (within 2 metres)	✓ single use[5]	✓ single use[5]	✗	✗	✓ single or sessional use[5,6]	✗	✓ risk assess single or sessional use[5,6,7]
	Operating theatre with possible or confirmed case(s)[3] – no AGPs[2]	✓ single use[5]	✓ single use[5]	✓ risk assess single use[5,7]	✗	✓ single or sessional use[5,6]	✗	✓ single or sessional use[5,6]
	Labour ward/area – 2nd/3rd stage labour vaginal delivery (no AGPs[2]) – possible or confirmed case[3]	✓ single use[5]	✓ single use[5]	✓ single use[2]	✗	✓ single or sessional use[5,6]	✗	✓ single or sessional use[5,6]
	Inpatient care to any individuals in the extremely vulnerable group undergoing shielding[8]	✓ single use[5]	✓ single use[5]	✗	✓ single use[5]	✗	✗	✗

Table 1

1. This may be single or reusable face/eye protection/full face visor or goggles.
2. The full list of aerosol generating procedures (AGPs) is within the COVID-19 IPC guidance [note APGs are undergoing a further review at present].
3. A case is any individual meeting case definition for a possible or confirmed case: https://www.gov.uk/government/publications/wuhan-novel-coronavirus-initial-investigation-of-possible-cases/investigation-and-initial-clinical-management-of-possible-cases-of-wuhan-novel-coronavirus-wn-cov-infection
4. Higher risk acute areas include: ICU/HDUs; ED resuscitation areas; wards with non-invasive ventilation; operating theatres; endoscopy units for upper Respiratory, ENT or upper GI endoscopy; and other clinical areas where AGPs are regularly performed.
5. Single use refers to disposal of PPE or decontamination of reusable items e.g. eye protection or respirator, after each patient and/or following completion of a procedure, task, or session; dispose or decontaminate reusable items after each patient contact as per Standard Infection Control Precautions (SICPs).
6. A session refers to a period of time where a healthcare worker is undertaking duties in a specific care setting/exposure environment e.g. on a ward round; providing ongoing care for inpatients. A session ends when the healthcare worker leaves the care setting/exposure environment. Sessional use should always be risk assessed and considered where there are high rates of hospital cases. PPE should be disposed of after each session or earlier if damaged, soiled, or uncomfortable.
7. Risk assessed use refers to utilising PPE when there is an anticipated/likely risk of contamination with splashes, droplets of blood or body fluids.
8. For explanation of shielding and definition of extremely vulnerable groups see guidance: https://www.gov.uk/government/publications/guidance-on-shielding-and-protecting-extremely-vulnerable-persons-from-covid-19/guidance-on-shielding-and-protecting-extremely-vulnerable-persons-from-covid-19

Patient use of PPE: In cohort wards, communal waiting areas and during transportation, it is recommended that suspected or confirmed cases wear a surgical face mask if this can be tolerated. The aim of this is to minimise the dispersal of respiratory secretions, reduce both direct transmission risk and environmental contamination. A surgical face mask should not be worn by patients if there is potential for their clinical care to be compromised (e.g. when receiving oxygen therapy).

© Crown copyright 2020. Public Health England Gateway number: 2019302. V1. 02-04-2020

Item 2 - The version of the document referred to in the guidance is no longer available at that link (https://assets.publishing.service.gov.uk/government/uploads/system/uploads/attachment_data/file/877728/T1_Recommended_PPE_for_healthcare_workers_by_secondary_care_clinical_context_poster.pdf), but can be obtained on The Internet Archive (https://web.archive.org/) and has been reproduced as an image here.

Public Health England | Academy of Medical Royal Colleges | GIG Cymru NHS Wales – Iechyd Cyhoeddus Cymru Public Health Wales | Health Protection Scotland | HSC Public Health Agency | NHS

Additional considerations, in addition to standard infection prevention and control precautions,

where there is sustained transmission of COVID-19, taking into account individual risk assessment for this new and emerging pathogen, NHS and independent sector

Setting	Context	Disposable Gloves	Disposable Plastic Apron	Disposable fluid-repellent coverall/ gown	Surgical mask	Fluid-resistant (Type IIR) surgical mask	Filtering face piece respirator	Eye/face protection[1]
Any setting	Direct patient/resident care assessing an individual that is not currently a possible or confirmed case[2] (within 2 metres)	✓ single use[3]	✓ single use[3]	✗	✗	✓ risk assess sessional use[4,5]	✗	✓ risk assess sessional use[4,5]
Any setting	Performing an aerosol generating procedure[6] on an individual that is not currently a possible or confirmed case[2]	✓ single use[3]	✗	✓ single use[3]	✗	✗	✓ single use[3]	✓ single use[3]

Table 4

1. This may be single or reusable face/eye protection/full face visor or goggles.
2. A case is any individual meeting case definition for a possible or confirmed case: https://www.gov.uk/government/publications/wuhan-novel-coronavirus-initial-investigation-of-possible-cases/investigation-and-initial-clinical-management-of-possible-cases-of-wuhan-novel-coronavirus-wn-cov-infection
3. Single use refers to disposal of PPE or decontamination of reusable items e.g. eye protection or respirator, after each patient and/or following completion of a procedure, task, or session; dispose or decontaminate reusable items after each patient contact as per Standard Infection Control Precautions (SICPs).
4. Risk assess refers to utilising PPE when there is an anticipated/likely risk of contamination with splashes, droplets of blood or body fluids. **Where staff consider there is a risk to themselves or the individuals they are caring for they should wear a fluid repellent surgical mask with or without eye protection as determined by the individual staff member for the care episode/single session.**
5. A single session refers to a period of time where a health care worker is undertaking duties in a specific care setting/exposure environment e.g. on a ward round; providing ongoing care for inpatients. A session ends when the health care worker leaves the care setting/exposure environment. Sessional use should always be risk assessed and consider the risk of infection to and from patients, residents and health and care workers where COVID-19 is circulating in the community and hospitals. PPE should be disposed of after each session or earlier if damaged, soiled, or uncomfortable.
6. The full list of aerosol generating procedures (AGPs) is within the IPC guidance [note APGs are undergoing a further review at present].

© Crown copyright 2020. Public Health England Gateway number: 2019302. V1. 02-04-2020

Item 3 - The version of the document referred to in the guidance is no longer available at that link (https://assets.publishing.service.gov.uk/government/uploads/system/uploads/attachment_data/file/877603/T4_Additional_considerations_of_COVID-19_poster.pdf)**, but the document can be obtained on The Internet Archive (**https://web.archive.org/**) and has been reproduced as an image here.**

Item 4
(https://www.gov.uk/government/publications/covid-19-management-of-exposed-healthcare-workers-and-patients-in-hospital-settings/covid-19-management-of-exposed-healthcare-workers-and-patients-in-hospital-settings) which was the original link is not now accessible. The text is reproduced below and the document can be obtained from the Internet Archive (https://web.archive.org/).

"Healthcare workers (HCWs) are vital for the functioning of the health system to ensure that we can treat patients appropriately. In addition, managers have a high level of skill in assessing whether individual staff are developing symptoms that would require exclusion from work. HCWs themselves are educated about prevention of the nosocomial spread of disease. It is a well-established practice for individuals not to come to work with respiratory tract infections.

1. Staff exposures

HCWs who come into contact with a COVID-19 patient or a patient suspected of having COVID-19 while not wearing personal protective equipment (PPE) can remain at work. This is because in most instances this will be a short-lived exposure, unlike exposure in a household setting that is ongoing.

HCWs should:

not attend work if they develop symptoms while at home (off-duty), and notify their line manager immediately

self-isolate and immediately inform their line manager if symptoms develop while at work

If the HCW's symptoms do not get better after 7 days, or their condition gets worse, they should speak to their occupational health department or use the NHS 111 online coronavirus service. If they do not have internet access, call NHS 111. For a medical emergency dial 999.

The current recommended PPE that must be worn when caring for COVID-19 patients is described in the infection prevention and control guidance.

These are guiding principles and there may need to be an individual risk assessment based on staff circumstances, for example for those who are immunocompromised.

2. Staff return to work criteria

Symptomatic staff can return to work:

on day 8 after the onset of symptoms if clinical improvement has occurred and they have been afebrile (not feverish) for 2 days

if a cough is the only persistent symptom on day 8, they can return to work (post-viral cough is known to persist for several weeks in some cases)

3. Patient exposures

In-patients who are known to have been exposed to a confirmed COVID-19 patient should be isolated or cohorted until their hospital admission ends, or until 14 days after last exposure.

If symptoms or signs consistent with COVID-19 occur in the 14 days after exposure then relevant diagnostic tests, including the COVID-19 test, should be performed.

On discharge, patients should be given written advice to stay at home and referred to the stay at home guidance if less than 14 days has elapsed since their exposure."

Appendix 5: Comorbidity definitions

The following ICD-10 definitions were used to define each comorbidity (British National Formulary definitions are included in our public github repository).

CIRCULATORY DISORDERS

Ischaemic heart disease	I20-I25
Other heart disease	I00-102
	I05-I09
	I10-I15
	I26-I28
	I30-I52
Cerebrovascular Disease	I60-I69
	G45 = Transient cerebral ischaemic attacks
	G46 = Vascular syndromes of brain in cerebrovascular diseases
Other circulatory system diseases	I70-I79 Diseases of arteries, arterioles and capillaries
	I80-I89 Diseases of veins, lymphatic vessels and lymph nodes, not elsewhere classified
	I95-I99 Other and unspecified disorders of the circulatory system
	Z958 presence of vascular implants and grafts
	Z959 presence of vascular implants and grafts NOS

Neurological diseases except inflammatory

Epilepsy	G40-G47 Episodic and paroxysmal disorders
Mono and polyneuropathies	G50-G59 Nerve, nerve root and plexus disorders
	G60-G64 Polyneuropathies and other disorders of the peripheral nervous system
Other neurological conditions	G10-G14 Systemic atrophies primarily affecting the central nervous system
	G20-G26 Extrapyramidal and movement disorders
	G30-G32 Other degenerative diseases of the nervous system
	G35-G37 Demyelinating diseases of the central nervous system
	G70-G73 Diseases of myoneural junction and muscle
	G80-G83 Cerebral palsy and other paralytic syndromes
	G90-G99 Other disorders of the nervous system

Respiratory diseases

Acute respiratory infections	J00-J06 Acute upper respiratory infections
	J09-J18 Influenza and pneumonia
	J20-J22 Other acute lower respiratory infections
Asthma	J45 Asthma

	J46 Status asthmaticus
Other Chronic lower respiratory disease	J40 Bronchitis, not specified as acute or chronic
	J41 Simple and mucopurulent chronic bronchitis<br>J42 Unspecified chronic bronchitis<br>J43 Emphysema<br>J44 Other chronic obstructive pulmonary disease<br>J47 Bronchiectasis<br>J60-J70 Lung diseases due to external agents<br>J80-J84 Other respiratory diseases principally affecting the interstitium<br>J85-J86 Suppurative and necrotic conditions of lower respiratory tract<br>J90-J94 Other diseases of pleura<br>J95-J99 Other diseases of the respiratory system<br>G473 Sleep apnoea
Tuberculosis	A15 Respiratory tuberculosis, bacteriologically and histologically confirmed<br>A16 Respiratory tuberculosis, not confirmed bacteriologically or histologically<br>A17 Tuberculosis of nervous system<br>A18 Tuberculosis of other organs<br>A19 Miliary tuberculosis
Connective tissue diseases	
Connective tissue disorder	M050 Felty syndrome
	M051 Rheumatoid lung disease<br>M052 Rheumatoid vasculitis<br>M053 Rheumatoid arthritis with involvement of other organs and systems<br>M058 Other seropositive rheumatoid arthritis<br>M059 Seropositive rheumatoid arthritis, unspecified<br>M060 Seronegative rheumatoid arthritis<br>M063 Rheumatoid nodule<br>M069 Rheumatoid arthritis, unspecified<br>M32 Systemic lupus erythematosus<br>M332 Polymyositis<br>M353 Polymyalgia rheumatica<br>M34 Systemic sclerosis
Decompensated liver disease	C22.0 hepatocellular carcinoma
	I85.0 Oesophageal varices<br>I98.3 Oesophageal varices<br>K70.4 Alcoholic hepatic failure<br>K72.0 Acute and subacute failure of the liver

	K72.1 Chronic hepatic failure
	K72.9 Hepatic coma
	K76.7 hepatorenal syndrome
	R18 Ascites
Kidney disease	
Advanced Chronic kidney disease or RRT	N18.3 Chronic kidney disease stage 3
	N18.4 Chronic kidney disease stage 4
	N18.5 Chronic kidney disease stage 5
	Z490 Care involving dialysis
	Z491 Care involving dialysis
	Z492 Care involving dialysis
	Z940 Kidney transplant status
	Z992 Dependence on renal dialysis
Other chronic kidney disease	N00-N08 Glomerular diseases
	N10-N16 Renal Tubulo-interstitial disease
	N18.1 Chronic kidney disease stage 1
	N18.2 Chronic kidney disease stage 2
	N18.9 Chronic kidney disease unspecified
Diabetes	
	E10 Type 1 diabetes mellitus
	E11 Type 2 diabetes mellitus
	E12 Malnutrition-related diabetes mellitus
	E13 Other specified diabetes mellitus
	E14 Unspecified diabetes mellitus
Malignant neoplasms	C00-C97
Lung cancers	C34 Malignant neoplasm of bronchus and lung
Blood cancers	C81-C96 Malignant neoplasms, stated or presumed to be primary, of lymphoid, haematopoietic and related tissue
Other cancers	Everything else in C00-97 except c34 and C81-C96
Immunological disease	
HIV	B20 –B23 Human immunodeficiency virus [HIV] disease
Certain disorders involving the immune system	D80-D89 Certain disorders involving the immune system
Sickle cell disease	
SCD	D57 Sickle-cell disorders
Cystic fibrosis	E84 Cystic fibrosis
Organ transplantation other than kidney	
Organ transplantation	Z941-Z949

Supplementary table A: Baseline characteristics of healthcare workers in patient facing roles stratified by front door, intensive care, specialties exposed to aerosolized generating procedures and other

	**Front door**	**ICU**	**AGP (non-ICU)**	**Other**
**Number, n (%)**	49411	1348	4231	35743
**Age, mean(sd)**	42.87 (11.65)	41.49 (9.9)	41.19 (10.72)	44.07 (11.4)
**Sex n(%)**				
Female	40820 (82.6%)	593 (44%)	3396 (80.3%)	27637 (77.3%)
**SIMD Quintile n(%)**				
1 (least deprived)	7560 (15.3%)	33 (2.4%)	238 (5.6%)	4567 (12.8%)
2	9524 (19.3%)	71 (5.3%)	466 (11%)	6395 (17.9%)
3	9961 (20.2%)	143 (10.6%)	811 (19.2%)	7263 (20.3%)
4	11150 (22.6%)	365 (27.1%)	1152 (27.2%)	8460 (23.7%)
5 (most deprived)	11216 (22.7%)	736 (54.6%)	1564 (37%)	9058 (25.3%)
**Ethnic group, n(%)**				
White	47775 (96.7%)	1200 (89%)	4064 (96.1%)	34283 (95.9%)
South Asian	1064 (2.2%)	121 (9%)	128 (3%)	1039 (2.9%)
Black	251 (0.5%)	7 (0.5%)	5 (0.1%)	174 (0.5%)
Chinese	160 (0.3%)	13 (1%)	18 (0.4%)	116 (0.3%)
Other	161 (0.3%)	7 (0.5%)	16 (0.4%)	131 (0.4%)
**Comorbidity, n(%)**				
Ischaemic heart disease	435 (6.7%)	7 (5.9%)	18 (5.4%)	355 (7.7%)
Other heart disease	1164 (17.9%)	25 (21%)	59 (17.7%)	812 (17.5%)
Other circulatory system diseases	828 (12.8%)	17 (14.3%)	32 (9.6%)	493 (10.6%)
Advanced chronic kidney disease	27 (0.4%)	1 (0.8%)	2 (0.6%)	20 (0.4%)
Asthma and chronic lower respiratory disease	1103 (17%)	20 (16.8%)	36 (10.8%)	714 (15.4%)
Neurological disorders	221 (3.4%)	7 (5.9%)	17 (5.1%)	144 (3.1%)
Decompensated liver disease	29 (0.4%)	0 (0%)	0 (0%)	25 (0.5%)
Malignant Neoplasms	1662 (25.6%)	40 (33.6%)	125 (37.4%)	1243 (26.8%)
Disorders of esophagus, stomach and duodenum	1259 (19.4%)	20 (16.8%)	47 (14.1%)	834 (18%)
Diabetes, type 1	337 (5.2%)	5 (4.2%)	29 (8.7%)	233 (5%)
Diabetes, type 2	960 (14.8%)	11 (9.2%)	27 (8.1%)	849 (18.3%)
Diabetes (type unknown)	74 (0.1%)	0 (0%)	1 (0%)	71 (0.2%)
**Comorbidity count, n(%)**				
0	42923 (86.9%)	1229 (91.2%)	3897 (92.1%)	31106 (87%)
1	5216 (10.6%)	99 (7.3%)	287 (6.8%)	3750 (10.5%)
>= 2	1272 (2.6%)	20 (1.5%)	47 (1.1%)	887 (2.5%)
**Immigration Status, n(%)**				

UK National	44130 (89.3%)	1306 (96.9%)	4214 (99.6%)	35482 (99.3%)
Non UK National	277 (0.6%)	42 (3.1%)	17 (0.4%)	261 (0.7%)
**Whole or Part Time, n(%)**				
Whole time	25376 (51.4%)	1218 (90.4%)	2227 (52.6%)	22360 (62.6%)
Part time	19031 (38.5%)	130 (9.6%)	2004 (47.4%)	13383 (37.4%)

Abbreviation: SIMD – Scottish Index of Multiple Deprivation; AGP – Aerosolised generating procedure, Immigration status and whole or part time status are not included in the GPCD database hence the percentages do not sum to 100% for these variables.

Supplementary table B: Model coefficients for healthcare workers

These tables show the complete set of regression models run as part of these analyses including sensitivity analyses. They are available in comma-separated (CSV) file formats in our public github repository (https://github.com/ChronicDiseaseEpi/hcw).

These coefficients and confidence intervals are provided in the interests of transparency to allow readers to judge the models presented and to view the additional models fitted as part of the wider analyses. Many of these models include sensitivity analyses, and so included groups with smaller numbers and/or included interaction terms. Therefore, the results presented in these tables should be interpreted with considerable caution. They should be examined alongside the R code used to fit the models (https://github.com/ChronicDiseaseEpi/hcw).

ps - penalised spline

Cox models

Model	Parameters	Estimate and 95% Confidence interval
agesex	ps(age10)3	0.80 (0.47-1.35)
agesex	ps(age10)4	0.64 (0.22-1.83)
agesex	ps(age10)5	0.54 (0.12-2.52)
agesex	ps(age10)6	0.60 (0.10-3.59)
agesex	ps(age10)7	0.85 (0.14-5.31)
agesex	ps(age10)8	1.35 (0.23-7.99)
agesex	ps(age10)9	1.78 (0.32-10.11)
agesex	ps(age10)10	1.97 (0.35-11.02)
agesex	ps(age10)11	2.32 (0.41-13.03)
agesex	ps(age10)12	2.60 (0.46-14.68)
agesex	ps(age10)13	2.88 (0.49-16.98)
agesex	ps(age10)14	3.19 (0.48-21.25)
agesex	male	1.59 (1.22-2.08)
agesex	rolepf_any	3.31 (2.13-5.13)
agesex	roleundetermined	1.64 (0.99-2.72)
ethnicsimd	ps(age10)3	0.82 (0.49-1.37)
ethnicsimd	ps(age10)4	0.66 (0.23-1.88)
ethnicsimd	ps(age10)5	0.57 (0.12-2.62)
ethnicsimd	ps(age10)6	0.64 (0.11-3.77)
ethnicsimd	ps(age10)7	0.92 (0.15-5.63)
ethnicsimd	ps(age10)8	1.46 (0.25-8.51)
ethnicsimd	ps(age10)9	1.92 (0.34-10.69)
ethnicsimd	ps(age10)10	2.09 (0.38-11.50)
ethnicsimd	ps(age10)11	2.42 (0.44-13.41)
ethnicsimd	ps(age10)12	2.64 (0.47-14.72)

ethnicsimd	ps(age10)13	2.84 (0.49-16.50)
ethnicsimd	ps(age10)14	3.03 (0.46-20.01)
ethnicsimd	male	1.64 (1.25-2.14)
ethnicsimd	rolepf_any	3.29 (2.12-5.10)
ethnicsimd	roleundetermined	1.54 (0.93-2.57)
ethnicsimd	white	1.13 (0.53-2.41)
ethnicsimd	simd	0.81 (0.73-0.88)
como	ps(age10)3	0.81 (0.48-1.36)
como	ps(age10)4	0.66 (0.23-1.86)
como	ps(age10)5	0.56 (0.12-2.59)
como	ps(age10)6	0.63 (0.11-3.70)
como	ps(age10)7	0.90 (0.15-5.49)
como	ps(age10)8	1.40 (0.24-8.18)
como	ps(age10)9	1.80 (0.32-10.05)
como	ps(age10)10	1.91 (0.35-10.51)
como	ps(age10)11	2.14 (0.39-11.89)
como	ps(age10)12	2.25 (0.40-12.58)
como	ps(age10)13	2.31 (0.39-13.50)
como	ps(age10)14	2.35 (0.35-15.66)
como	male	1.56 (1.19-2.04)
como	rolepf_any	3.30 (2.13-5.13)
como	roleundetermined	1.55 (0.93-2.59)
como	white	1.21 (0.57-2.60)
como	simd	0.82 (0.75-0.90)
como	como_count	1.23 (1.03-1.46)
como	t2dm	2.08 (1.23-3.52)
ethnicalone	ps(age10)3	0.82 (0.50-1.34)
ethnicalone	ps(age10)4	0.67 (0.25-1.80)
ethnicalone	ps(age10)5	0.57 (0.13-2.44)
ethnicalone	ps(age10)6	0.62 (0.11-3.40)
ethnicalone	ps(age10)7	0.86 (0.15-4.91)
ethnicalone	ps(age10)8	1.33 (0.24-7.24)
ethnicalone	ps(age10)9	1.74 (0.33-9.08)
ethnicalone	ps(age10)10	1.91 (0.37-9.85)
ethnicalone	ps(age10)11	2.20 (0.42-11.38)
ethnicalone	ps(age10)12	2.34 (0.45-12.19)
ethnicalone	ps(age10)13	2.44 (0.45-13.27)
ethnicalone	ps(age10)14	2.53 (0.41-15.64)
ethnicalone	male	1.61 (1.23-2.11)
ethnicalone	white	1.12 (0.53-2.39)
occupation	ps(age10)3	0.81 (0.48-1.36)
occupation	ps(age10)4	0.66 (0.23-1.86)
occupation	ps(age10)5	0.56 (0.12-2.58)
occupation	ps(age10)6	0.62 (0.11-3.69)
occupation	ps(age10)7	0.89 (0.15-5.47)

occupation	ps(age10)8	1.40 (0.24-8.16)
occupation	ps(age10)9	1.79 (0.32-10.03)
occupation	ps(age10)10	1.90 (0.34-10.48)
occupation	ps(age10)11	2.13 (0.38-11.88)
occupation	ps(age10)12	2.25 (0.40-12.61)
occupation	ps(age10)13	2.32 (0.40-13.57)
occupation	ps(age10)14	2.37 (0.36-15.80)
occupation	male	1.59 (1.21-2.09)
occupation	rolepf_any	3.00 (1.68-5.36)
occupation	roleundetermined	1.53 (0.91-2.56)
occupation	white	1.23 (0.57-2.62)
occupation	simd	0.82 (0.74-0.90)
occupation	como_count	1.23 (1.03-1.46)
occupation	t2dm	2.09 (1.23-3.53)
occupation	nurs_med_ahp	1.12 (0.72-1.73)
parttime	ps(age10)3	0.83 (0.49-1.42)
parttime	ps(age10)4	0.69 (0.24-2.01)
parttime	ps(age10)5	0.61 (0.13-2.90)
parttime	ps(age10)6	0.71 (0.12-4.30)
parttime	ps(age10)7	1.03 (0.16-6.53)
parttime	ps(age10)8	1.62 (0.27-9.74)
parttime	ps(age10)9	2.06 (0.36-11.80)
parttime	ps(age10)10	2.16 (0.38-12.23)
parttime	ps(age10)11	2.45 (0.43-13.99)
parttime	ps(age10)12	2.64 (0.46-15.17)
parttime	ps(age10)13	2.78 (0.46-16.79)
parttime	ps(age10)14	2.90 (0.42-20.12)
parttime	male	1.47 (1.10-1.96)
parttime	rolepf_any	3.06 (1.73-5.43)
parttime	roleundetermined	1.60 (0.96-2.69)
parttime	white	1.21 (0.57-2.59)
parttime	simd	0.83 (0.75-0.91)
parttime	como_count	1.23 (1.03-1.46)
parttime	t2dm	2.05 (1.21-3.46)
parttime	nurs_med_ahp	1.13 (0.74-1.74)
parttime	part_timep	0.75 (0.56-1.00)
parttime	part_timegeneral practice	0.44 (0.16-1.18)
gradeafc	ps(age10)3	0.75 (0.45-1.27)
gradeafc	ps(age10)4	0.56 (0.20-1.61)
gradeafc	ps(age10)5	0.45 (0.10-2.08)
gradeafc	ps(age10)6	0.47 (0.08-2.86)
gradeafc	ps(age10)7	0.65 (0.10-4.13)
gradeafc	ps(age10)8	1.04 (0.17-6.26)
gradeafc	ps(age10)9	1.43 (0.25-8.13)
gradeafc	ps(age10)10	1.61 (0.29-8.97)

gradeafc	ps(age10)11	1.84 (0.33-10.35)
gradeafc	ps(age10)12	1.97 (0.35-11.11)
gradeafc	ps(age10)13	2.09 (0.35-12.48)
gradeafc	ps(age10)14	2.23 (0.32-15.44)
gradeafc	male	1.32 (0.96-1.82)
gradeafc	rolepf_any	2.94 (1.59-5.45)
gradeafc	roleundetermined	1.66 (0.99-2.79)
gradeafc	white	1.30 (0.41-4.09)
gradeafc	simd	0.80 (0.72-0.90)
gradeafc	como_count	1.26 (1.06-1.50)
gradeafc	t2dm	2.00 (1.18-3.42)
gradeafc	nurs_med_ahp	1.12 (0.71-1.78)
gradeafc	part_timep	0.67 (0.50-0.91)
gradeafc	part_timegeneral practice	
gradeafc	grade5-7	1.03 (0.75-1.41)
gradeafc	grade8+	1.18 (0.53-2.62)
grademed	ps(age)3	3.42 (2.23-5.24)
grademed	ps(age)4	11.62 (4.94-27.37)
grademed	ps(age)5	31.68 (7.92-126.70)
grademed	ps(age)6	66.57 (7.79-568.67)
grademed	ps(age)7	134.16 (12.32-1460.40)
grademed	ps(age)8	172.11 (18.98-1560.42)
grademed	ps(age)9	121.55 (13.98-1056.54)
grademed	ps(age)10	70.52 (7.57-656.66)
grademed	ps(age)11	56.89 (5.42-597.19)
grademed	ps(age)12	61.05 (4.25-876.14)
grademed	ps(age)13	70.34 (2.37-2088.26)
grademed	ps(age)14	78.64 (1.05-5874.62)
grademed	male	2.58 (1.06-6.29)
grademed	simd	1.07 (0.68-1.68)
grademed	gradespecialty_assoc_spec	0.83 (0.11-6.47)
grademed	gradetraining_grade	2.02 (0.71-5.77)
pt	ps(age10)3	0.83 (0.49-1.42)
pt	ps(age10)4	0.69 (0.24-2.01)
pt	ps(age10)5	0.61 (0.13-2.90)
pt	ps(age10)6	0.71 (0.12-4.30)
pt	ps(age10)7	1.03 (0.16-6.53)
pt	ps(age10)8	1.62 (0.27-9.74)
pt	ps(age10)9	2.06 (0.36-11.80)
pt	ps(age10)10	2.16 (0.38-12.23)
pt	ps(age10)11	2.45 (0.43-13.99)
pt	ps(age10)12	2.64 (0.46-15.17)
pt	ps(age10)13	2.78 (0.46-16.79)
pt	ps(age10)14	2.90 (0.42-20.12)
pt	male	1.47 (1.10-1.96)

pt	rolepf_any	3.06 (1.73-5.43)
pt	roleundetermined	1.60 (0.96-2.69)
pt	white	1.21 (0.57-2.59)
pt	simd	0.83 (0.75-0.91)
pt	como_count	1.23 (1.03-1.46)
pt	t2dm	2.05 (1.21-3.46)
pt	nurs_med_ahp	1.13 (0.74-1.74)
pt	part_timep	0.75 (0.56-1.00)
pt	part_timegeneral practice	0.44 (0.16-1.18)
immigration	ps(age10)3	0.82 (0.49-1.40)
immigration	ps(age10)4	0.68 (0.23-1.95)
immigration	ps(age10)5	0.59 (0.12-2.77)
immigration	ps(age10)6	0.66 (0.11-4.02)
immigration	ps(age10)7	0.94 (0.15-5.90)
immigration	ps(age10)8	1.49 (0.25-8.87)
immigration	ps(age10)9	1.96 (0.35-11.14)
immigration	ps(age10)10	2.10 (0.37-11.75)
immigration	ps(age10)11	2.38 (0.42-13.39)
immigration	ps(age10)12	2.55 (0.45-14.49)
immigration	ps(age10)13	2.69 (0.45-16.09)
immigration	ps(age10)14	2.82 (0.41-19.36)
immigration	male	1.42 (1.06-1.91)
immigration	rolepf_any	3.09 (1.74-5.48)
immigration	roleundetermined	1.60 (0.96-2.69)
immigration	simd	0.82 (0.74-0.90)
immigration	como_count	1.24 (1.04-1.47)
immigration	t2dm	2.00 (1.18-3.37)
immigration	nurs_med_ahp	1.11 (0.72-1.71)
immigration	part_timep	0.74 (0.56-1.00)
immigration	part_timegeneral practice	
immigration	immigration	2.27 (0.73-7.11)
ageroleinter	ps(age10)3	0.88 (0.51-1.50)
ageroleinter	ps(age10)4	0.77 (0.26-2.25)
ageroleinter	ps(age10)5	0.72 (0.15-3.44)
ageroleinter	ps(age10)6	0.88 (0.14-5.46)
ageroleinter	ps(age10)7	1.37 (0.21-9.10)
ageroleinter	ps(age10)8	2.27 (0.34-15.20)
ageroleinter	ps(age10)9	3.04 (0.45-20.57)
ageroleinter	ps(age10)10	3.35 (0.47-23.79)
ageroleinter	ps(age10)11	3.98 (0.52-30.32)
ageroleinter	ps(age10)12	4.45 (0.55-36.15)
ageroleinter	ps(age10)13	4.81 (0.53-43.73)
ageroleinter	ps(age10)14	5.18 (0.47-56.64)
ageroleinter	male	1.48 (1.11-1.98)
ageroleinter	rolepf_any	8.34 (0.65-107.64)

ageroleinter	roleundetermined	2.10 (0.11-40.94)
ageroleinter	white	1.21 (0.57-2.59)
ageroleinter	simd	0.83 (0.75-0.91)
ageroleinter	como_count	1.23 (1.03-1.46)
ageroleinter	t2dm	2.04 (1.21-3.46)
ageroleinter	nurs_med_ahp	1.13 (0.74-1.74)
ageroleinter	part_timep	0.75 (0.56-1.00)
ageroleinter	part_timegeneral practice	0.44 (0.16-1.17)
ageroleinter	rolenpf:age10	1.05 (0.60-1.86)
ageroleinter	rolepf_any:age10	0.87 (0.61-1.23)
ageroleinter	roleundetermined:age10	
sexroleinter	ps(age10)3	0.84 (0.49-1.42)
sexroleinter	ps(age10)4	0.70 (0.24-2.02)
sexroleinter	ps(age10)5	0.62 (0.13-2.93)
sexroleinter	ps(age10)6	0.72 (0.12-4.37)
sexroleinter	ps(age10)7	1.06 (0.17-6.64)
sexroleinter	ps(age10)8	1.66 (0.28-9.91)
sexroleinter	ps(age10)9	2.10 (0.37-12.02)
sexroleinter	ps(age10)10	2.21 (0.39-12.46)
sexroleinter	ps(age10)11	2.51 (0.44-14.23)
sexroleinter	ps(age10)12	2.69 (0.47-15.36)
sexroleinter	ps(age10)13	2.81 (0.47-16.96)
sexroleinter	ps(age10)14	2.94 (0.43-20.28)
sexroleinter	male	1.13 (0.42-3.05)
sexroleinter	rolepf_any	2.96 (1.55-5.66)
sexroleinter	roleundetermined	1.17 (0.61-2.26)
sexroleinter	white	1.20 (0.56-2.55)
sexroleinter	simd	0.83 (0.75-0.91)
sexroleinter	como_count	1.23 (1.03-1.46)
sexroleinter	t2dm	2.04 (1.21-3.46)
sexroleinter	nurs_med_ahp	1.14 (0.72-1.79)
sexroleinter	part_timep	0.75 (0.57-1.01)
sexroleinter	part_timegeneral practice	0.45 (0.17-1.21)
sexroleinter	male:rolepf_any	1.17 (0.41-3.34)
sexroleinter	male:roleundetermined	2.28 (0.70-7.47)
sexrolemale	ps(age10)3	2.85 (1.81-4.49)
sexrolemale	ps(age10)4	8.13 (3.28-20.14)
sexrolemale	ps(age10)5	22.86 (5.76-90.68)
sexrolemale	ps(age10)6	56.08 (7.76-405.18)
sexrolemale	ps(age10)7	118.86 (10.20-1384.81)
sexrolemale	ps(age10)8	218.31 (16.95-2811.59)
sexrolemale	ps(age10)9	255.84 (22.29-2937.00)
sexrolemale	ps(age10)10	202.06 (19.18-2128.91)
sexrolemale	ps(age10)11	207.21 (19.74-2175.31)
sexrolemale	ps(age10)12	250.44 (23.72-2643.78)

sexrolemale	ps(age10)13	347.01 (29.82-4037.78)
sexrolemale	ps(age10)14	510.15 (30.38-8567.58)
sexrolemale	rolepf_any	4.18 (1.49-11.70)
sexrolemale	roleundetermined	2.79 (1.02-7.60)
sexrolemale	white	0.94 (0.37-2.36)
sexrolemale	simd	0.95 (0.80-1.12)
sexrolemale	como_count	1.11 (0.83-1.49)
sexrolemale	t2dm	1.99 (0.87-4.56)
sexrolemale	nurs_med_ahp	0.84 (0.46-1.52)
sexrolemale	part_timep	0.89 (0.47-1.71)
sexrolemale	part_timegeneral practice	0.63 (0.20-2.05)
sexrolefemale	ps(age10)3	0.71 (0.40-1.25)
sexrolefemale	ps(age10)4	0.50 (0.16-1.56)
sexrolefemale	ps(age10)5	0.38 (0.07-2.00)
sexrolefemale	ps(age10)6	0.39 (0.06-2.68)
sexrolefemale	ps(age10)7	0.53 (0.07-3.69)
sexrolefemale	ps(age10)8	0.77 (0.12-5.05)
sexrolefemale	ps(age10)9	0.99 (0.16-6.18)
sexrolefemale	ps(age10)10	1.18 (0.19-7.18)
sexrolefemale	ps(age10)11	1.40 (0.23-8.61)
sexrolefemale	ps(age10)12	1.39 (0.22-8.71)
sexrolefemale	ps(age10)13	1.24 (0.18-8.43)
sexrolefemale	ps(age10)14	1.08 (0.13-8.67)
sexrolefemale	rolepf_any	2.27 (1.04-4.96)
sexrolefemale	roleundetermined	1.10 (0.56-2.16)
sexrolefemale	white	1.86 (0.46-7.59)
sexrolefemale	simd	0.78 (0.69-0.88)
sexrolefemale	como_count	1.29 (1.04-1.61)
sexrolefemale	t2dm	2.21 (1.13-4.32)
sexrolefemale	nurs_med_ahp	1.53 (0.78-2.99)
sexrolefemale	part_timep	0.75 (0.54-1.02)
sexrolefemale	part_timegeneral practice	0.24 (0.03-1.74)
subrole	ps(age10)3	0.70 (0.18-2.76)
subrole	ps(age10)4	0.49 (0.05-4.72)
subrole	ps(age10)5	0.37 (0.03-5.40)
subrole	ps(age10)6	0.40 (0.03-6.25)
subrole	ps(age10)7	0.64 (0.04-9.43)
subrole	ps(age10)8	1.10 (0.08-15.54)
subrole	ps(age10)9	1.39 (0.10-19.31)
subrole	ps(age10)10	1.27 (0.09-17.66)
subrole	ps(age10)11	1.35 (0.10-18.80)
subrole	ps(age10)12	1.67 (0.12-23.39)
subrole	ps(age10)13	1.89 (0.13-27.92)
subrole	ps(age10)14	2.05 (0.10-40.87)
subrole	male	1.41 (0.98-2.04)

subrole	role_subpf_front	2.09 (1.49-2.94)
subrole	role_subpf_resp_oro_agp	1.91 (0.90-4.07)
subrole	role_subpf_icu	1.22 (0.29-5.09)
subrole	white	0.96 (0.44-2.06)
subrole	simd	0.86 (0.77-0.96)
subrole	como_count	1.22 (0.96-1.55)
subrole	t2dm	2.25 (1.23-4.12)
subrole	nurs_med_ahp	1.08 (0.57-2.03)
subrole	part_timep	0.69 (0.50-0.97)
subrole	part_timegeneral practice	0.31 (0.11-0.87)

Conditional logistic regression models

Model	Parameters	Estimate and 95% confidence interval
agesex	rolepopulation	0.92 (0.59-1.42)
ethnicsimd	rolepopulation	0.90 (0.58-1.40)
ethnicsimd	white	1.47 (0.86-2.50)
ethnicsimd	simd	0.87 (0.83-0.91)
como	rolepopulation	0.81 (0.52-1.26)
como	white	1.70 (1.00-2.92)
como	simd	0.91 (0.87-0.95)
como	como_count	1.76 (1.68-1.84)

Supplementary table C: Risk of COVID-19 hospitalization within healthcare workers comparing occupational role (nursing and midwifery occupational role as the referent)

	Nursing and midwifery	Medical, other	Medical, general practice	Allied health professional	Support services	Administrative services	Other
**Hospitalized (n)**	125	20	4	27	30	19	18
**Total population (n)**	64560	11513	5004	14046	16661	28532	18129
**Risk (%)**	0.19	0.17	0.08	0.19	0.18	0.07	0.1
**Model 1, Age, sex and role**	1	0.93 (0.57-1.52)	0.37 (0.14-0.99)	1.05 (0.69-1.59)	1.28 (0.78-2.09)	0.76 (0.25-2.30)	0.88 (0.49-1.58)
**Model 2, as model 1 plus socioeconomic deprivation and ethnicity**	1	1.28 (0.76-2.17)	0.49 (0.18-1.32)	1.18 (0.78-1.81)	1.06 (0.64-1.75)	0.71 (0.23-2.15)	0.89 (0.50-1.61)
**Model 3, as model 2, plus comorbidity**	1	1.34 (0.79-2.26)	0.53 (0.20-1.40)	1.22 (0.80-1.86)	1.03 (0.62-1.71)	0.70 (0.23-2.12)	0.90 (0.50-1.62)
**Model 4, as model 4 plus part time status**	1	1.31 (0.77-2.21)	0.48 (0.18-1.28)	1.23 (0.80-1.87)	1.07 (0.65-1.76)	0.69 (0.23-2.08)	0.87 (0.48-1.55)

Supplementary table D: Model coefficients for household members

These tables show the complete set of regression models run as part of these analyses including sensitivity analyses. They are available in comma-separated (CSV) file formats in our public github repository (https://github.com/ChronicDiseaseEpi/hcw). They should be interpreted with the same caveats noted in Supplementary table 2

Cox models

Model	Parameters	Estimate and 95% confidence interval
agesex	ps(age10)3	1.02 (0.37-2.82)
agesex	ps(age10)4	1.13 (0.17-7.70)
agesex	ps(age10)5	1.60 (0.14-18.16)
agesex	ps(age10)6	3.15 (0.24-40.75)
agesex	ps(age10)7	8.41 (0.68-103.66)
agesex	ps(age10)8	21.21 (1.84-245.13)
agesex	ps(age10)9	33.21 (2.92-378.18)
agesex	ps(age10)10	33.08 (2.88-380.55)
agesex	ps(age10)11	37.48 (3.17-442.90)
agesex	ps(age10)12	46.35 (3.62-593.22)
agesex	ps(age10)13	51.36 (3.55-742.60)
agesex	ps(age10)14	55.87 (3.25-959.51)
agesex	male	2.06 (1.35-3.13)
agesex	rolepf_any	1.81 (1.11-2.94)
agesex	roleundetermined	1.68 (0.95-2.95)
agesex_kids	ps(age10)3	0.02 (0.00-0.08)
agesex_kids	ps(age10)4	0.00 (0.00-0.01)
agesex_kids	ps(age10)5	0.00 (0.00-0.00)
agesex_kids	ps(age10)6	0.00 (0.00-0.00)
agesex_kids	ps(age10)7	0.00 (0.00-0.00)
agesex_kids	ps(age10)8	0.00 (0.00-0.03)
agesex_kids	ps(age10)9	0.01 (0.00-0.21)
agesex_kids	ps(age10)10	0.00 (0.00-0.10)
agesex_kids	ps(age10)11	0.00 (0.00-0.14)
agesex_kids	ps(age10)12	0.00 (0.00-0.06)
agesex_kids	ps(age10)13	0.00 (0.00-0.01)
agesex_kids	ps(age10)14	0.00 (0.00-0.00)
agesex_kids	male	3.80 (0.44-32.73)
agesex_kids	rolepf_any	0.36 (0.06-2.15)
agesex_kids	roleundetermined	NA (NA- NA)
agesex_gt65	ps(age10)3	2.07 (0.58-7.43)
agesex_gt65	ps(age10)4	4.04 (0.38-43.41)
agesex_gt65	ps(age10)5	4.35 (0.32-59.32)
agesex_gt65	ps(age10)6	4.03 (0.34-47.78)
agesex_gt65	ps(age10)7	4.66 (0.41-52.47)
agesex_gt65	ps(age10)8	5.36 (0.53-54.04)
agesex_gt65	ps(age10)9	9.44 (0.85-105.32)
agesex_gt65	ps(age10)10	12.17 (0.99-149.71)
agesex_gt65	ps(age10)11	6.66 (0.54-82.38)
agesex_gt65	ps(age10)12	2.24 (0.20-25.69)
agesex_gt65	ps(age10)13	0.69 (0.06-8.00)
agesex_gt65	ps(age10)14	0.21 (0.02-2.79)
agesex_gt65	male	1.60 (0.62-4.13)

agesex_gt65	rolepf_any	1.81 (0.54-6.06)
agesex_gt65	roleundetermined	1.42 (0.33-6.17)
ethnicsimd	ps(age10)3	1.01 (0.37-2.81)
ethnicsimd	ps(age10)4	1.11 (0.16-7.64)
ethnicsimd	ps(age10)5	1.57 (0.14-17.92)
ethnicsimd	ps(age10)6	3.08 (0.24-40.15)
ethnicsimd	ps(age10)7	8.26 (0.66-102.59)
ethnicsimd	ps(age10)8	20.93 (1.80-243.80)
ethnicsimd	ps(age10)9	32.66 (2.84-375.31)
ethnicsimd	ps(age10)10	32.15 (2.77-373.40)
ethnicsimd	ps(age10)11	36.07 (3.02-430.53)
ethnicsimd	ps(age10)12	44.39 (3.43-575.25)
ethnicsimd	ps(age10)13	49.07 (3.34-720.75)
ethnicsimd	ps(age10)14	53.27 (3.04-932.66)
ethnicsimd	male	2.07 (1.36-3.15)
ethnicsimd	rolepf_any	1.79 (1.10-2.92)
ethnicsimd	roleundetermined	1.63 (0.93-2.87)
ethnicsimd	white	0.80 (0.36-1.81)
ethnicsimd	simd	0.92 (0.81-1.04)
ethnicalone	ps(age10)3	1.01 (0.37-2.81)
ethnicalone	ps(age10)4	1.11 (0.16-7.65)
ethnicalone	ps(age10)5	1.57 (0.14-18.01)
ethnicalone	ps(age10)6	3.09 (0.24-40.37)
ethnicalone	ps(age10)7	8.22 (0.66-102.20)
ethnicalone	ps(age10)8	20.60 (1.77-240.07)
ethnicalone	ps(age10)9	31.99 (2.79-367.45)
ethnicalone	ps(age10)10	31.51 (2.72-365.04)
ethnicalone	ps(age10)11	35.56 (2.99-422.36)
ethnicalone	ps(age10)12	44.20 (3.43-569.03)
ethnicalone	ps(age10)13	49.42 (3.39-721.22)
ethnicalone	ps(age10)14	54.26 (3.11-945.06)
ethnicalone	male	2.04 (1.34-3.10)
ethnicalone	white	0.80 (0.35-1.80)
como	ps(age10)3	1.01 (0.36-2.85)
como	ps(age10)4	1.11 (0.16-7.84)
como	ps(age10)5	1.57 (0.13-18.49)
como	ps(age10)6	3.08 (0.23-40.97)
como	ps(age10)7	7.95 (0.63-100.17)
como	ps(age10)8	18.08 (1.53-213.67)
como	ps(age10)9	22.66 (1.94-265.42)
como	ps(age10)10	17.51 (1.48-207.79)
como	ps(age10)11	18.11 (1.48-221.04)
como	ps(age10)12	24.75 (1.82-336.12)
como	ps(age10)13	32.79 (2.00-536.27)
como	ps(age10)14	42.88 (2.02-908.35)
como	male	1.94 (1.27-2.96)
como	rolepf_any	1.78 (1.10-2.90)
como	roleundetermined	1.60 (0.91-2.82)
como	white	0.92 (0.41-2.07)
como	simd	0.97 (0.85-1.09)
como	como_count	1.60 (1.27-2.01)
como	heart_other_any1	0.60 (0.30-1.21)
como	t2dm	2.02 (1.22-3.37)

como	unkdm	4.84 (1.46-16.00)
como	ckd_any1	4.39 (1.40-13.77)
como	oad_any1	1.13 (0.52-2.46)
occupation	ps(age10)3	0.99 (0.35-2.80)
occupation	ps(age10)4	1.06 (0.15-7.59)
occupation	ps(age10)5	1.48 (0.12-17.76)
occupation	ps(age10)6	2.91 (0.21-39.81)
occupation	ps(age10)7	7.57 (0.58-98.17)
occupation	ps(age10)8	16.78 (1.38-203.81)
occupation	ps(age10)9	20.55 (1.71-247.11)
occupation	ps(age10)10	16.10 (1.32-196.25)
occupation	ps(age10)11	17.20 (1.37-215.39)
occupation	ps(age10)12	24.43 (1.76-339.99)
occupation	ps(age10)13	33.70 (2.01-564.21)
occupation	ps(age10)14	45.90 (2.12-994.59)
occupation	male	1.90 (1.25-2.88)
occupation	rolepf_any	1.60 (0.53-4.82)
occupation	roleundetermined	1.13 (0.36-3.53)
occupation	white	0.72 (0.32-1.64)
occupation	simd	1.03 (0.90-1.16)
occupation	como_count	1.60 (1.27-2.01)
occupation	heart_other_any1	0.60 (0.30-1.20)
occupation	t2dm	1.96 (1.17-3.27)
occupation	unkdm	4.81 (1.45-15.93)
occupation	ckd_any1	4.37 (1.40-13.71)
occupation	oad_any1	1.12 (0.52-2.42)
occupation	job_family_grpallied heal	2.03 (0.62-6.63)
occupation	job_family_grpadministrat	2.54 (0.57-11.34)
occupation	job_family_grpOther	2.92 (0.84-10.11)
occupation	job_family_grpnursing and	3.39 (1.23-9.32)
occupation	job_family_grpsupport ser	5.00 (1.47-17.04)
pt	ps(age10)3	0.96 (0.34-2.72)
pt	ps(age10)4	1.00 (0.14-7.14)
pt	ps(age10)5	1.37 (0.11-16.29)
pt	ps(age10)6	2.67 (0.20-36.31)
pt	ps(age10)7	6.97 (0.54-90.07)
pt	ps(age10)8	15.30 (1.27-184.98)
pt	ps(age10)9	18.66 (1.56-223.73)
pt	ps(age10)10	14.65 (1.20-178.20)
pt	ps(age10)11	15.67 (1.25-195.84)
pt	ps(age10)12	22.47 (1.61-313.17)
pt	ps(age10)13	31.39 (1.86-530.86)
pt	ps(age10)14	43.29 (1.96-958.58)
pt	male	2.02 (1.33-3.07)
pt	rolepf_any	1.60 (0.53-4.76)
pt	roleundetermined	1.19 (0.39-3.66)
pt	white	0.74 (0.33-1.69)
pt	simd	1.03 (0.91-1.17)
pt	como_count	1.59 (1.26-2.01)
pt	heart_other_any1	0.60 (0.30-1.20)
pt	t2dm	1.96 (1.18-3.28)
pt	unkdm	4.79 (1.45-15.85)

pt	ckd_any1	4.44 (1.40-14.04)
pt	oad_any1	1.12 (0.51-2.43)
pt	job_family_grpallied heal	1.87 (0.50-6.98)
pt	job_family_grpadministrat	2.31 (0.48-11.07)
pt	job_family_grpOther	2.48 (0.65-9.48)
pt	job_family_grpnursing and	3.11 (0.97-9.96)
pt	job_family_grpsupport ser	4.75 (1.27-17.76)
pt	part_timep	0.70 (0.49-1.01)
pt	part_timegeneral practice	0.55 (0.06-5.39)

Conditional logistic regression models

Model	Parameters	Estimate and 95% Confidence Interval
agesex	rolepopulation	0.87 (0.50-1.49)
ethnicsimd	rolepopulation	0.83 (0.48-1.43)
ethnicsimd	white	1.49 (0.88-2.54)
ethnicsimd	simd	0.87 (0.84-0.91)
como	rolepopulation	0.86 (0.49-1.51)
como	white	1.74 (1.02-2.98)
como	simd	0.92 (0.88-0.96)
como	como_count	1.76 (1.68-1.84)
wa	hhold_wa	2.23 (1.78-2.79)
modkids	rolepopulation	0.10 (0.01-1.60)
modolder	rolepopulation	1.99 (0.62-6.41)

Supplementary figure A

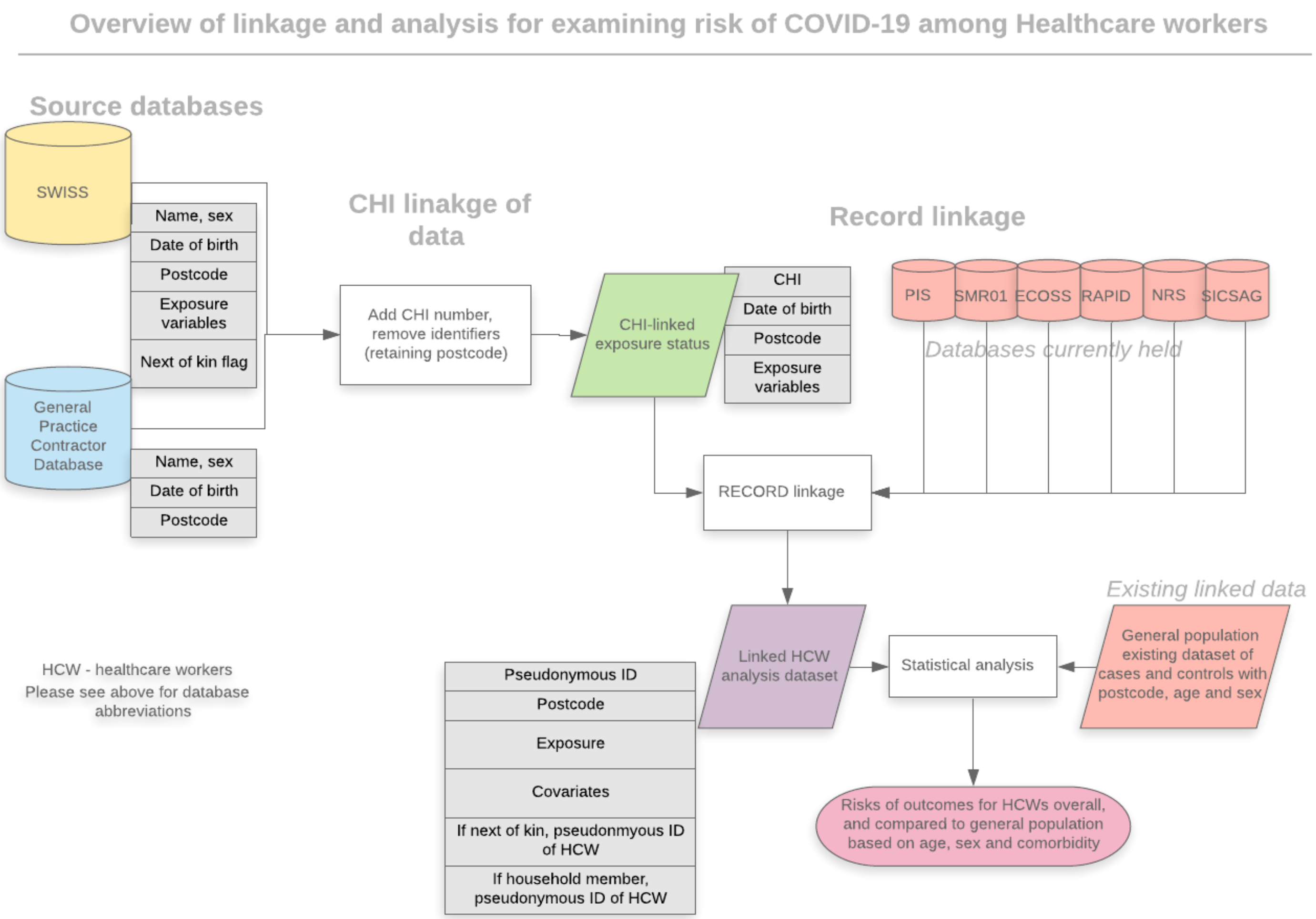

Supplementary figure B

	Other Scottish population	Healthcare worker data	Household members
Non cases	Non-sampled Scottish population (n = 5,009,368)<br>Randomly sampled* (n=59,620)	Healthcare worker population (aged 18-65 years) (n=158,202)	Household member population (all ages) (n=229,764)
Cases (COVID-19) hospitalisations	All cases of COVID-19 hospitalization in Scotland* (n=5,962)	All cases of COVID-19 hospitalization in healthcare workers* (n=243)	All cases of COVID-19 hospitalization in household members* (n=141)
Total	n=5,074,950	n=158,445	n=229,905

Explanatory notes

- Age, sex and Scottish index of multiple deprivation, and outcome data was available for all Scottish residents. Additional covariate information was available for those Scottish residents indicated in the green boxes.
- The total Scottish population, based on 2019 mid-year estimates was 5,463,300.
- The total number of cases in the Scottish population was 6,346
- *this population forms part of the REACT-COVID-19 nested case control study. As such clinical information was available on **all** COVID-19 hospitalizations in Scotland including those in healthcare workers and their households as well as a random sample of controls.
