# Supplementary material for: Risk of hospital admission with coronavirus disease 2019 in healthcare workers and their households: nationwide linkage cohort study
Source: BMJ. 2020 Oct 28;371:m3582. doi: 10.1136/bmj.m3582 (PMC7591828; doi:10.1136/bmj.m3582)
Supplement: Supplementary file 2 — Web appendix: Statistical analysis plan [file shaa060836.ww2.pdf]

Dynamic statistical analysis plan for study of the risk of COVID-19 in NHS Staff

Background

By the nature of their work, many healthcare workers (HCWs) have greater exposure to SARS-CoV2 than the general population. To inform HCWs and make optimal decisions about minimising risk, we need to know whether and how this increased exposure translates to an increased risk of COVID19-related (i) hospitalisation, (ii) mechanical ventilation and other forms of intensive care and (iii) death. We need to know both the overall risk, and the risk according to the HCWs role (since HCW is usually defined to include individuals in supporting roles within healthcare organisations as well as those providing direct care) as well as their age, sex and the presence/absence of any underlying long-term conditions.

To guide the monitoring of HCWs as part of the wider COVID19 prevention strategy it is also important for health services to know rates of testing as well as rates of testing positive among different types of HCWs working in different settings. Finally, to inform infection transmission models, it is also necessary to estimate how much transmission is occurring through HCWs.

Within Scotland, it is possible to produce these estimates for many HCWs because there exists a national database used to help manage the NHS workforce, the Scottish Workforce Information Standard System (SWISS). SWISS includes details of each staff member's role, grade, occupation, service area and working location, alongside their name, date of birth and postcode. SWISS covers all directly employed NHS staff, around 150,000 individuals. In addition, Public Health Scotland holds the General Practice Contractor Database (GPCD) which, includes the name, date of birth, sex and postcode of all general practice (GP) partners as well as salaried GPs. Therefore, Scotland has comprehensive data on NHS staff. The SWISS and GPCD database can be linked to healthcare records held within Public Health Scotland in order to address the following aims and objectives.

Aims and objectives

Aims

1 To estimate the risk of COVID19 among HCWs and members of their households.

2 To estimate rates of SARS-CoV2 testing among HCWs

3 To produce summary statistics on transmission among HCWs

Objectives

1.1 To estimate the risk of hospitalisation for COVID19 among HCWs

1.2 To estimate the risk of intensive care unit admission for COVID19 among HCWs

1.3 To estimate 1.2 and 1.3 according to role (see below), professional grouping (eg medical, nursing, AHP etc), seniority grade, age, sex and common underlying conditions

1.4 To estimate 1.1-1.3 for household members of HCWs

2.1 To estimate rates of any testing and testing positive among HCWs across role, occupation, grade, age, sex and common underlying conditions

3.1 To produce summary statistics on the number of infections among next of kin and household members of HCWs.

Methods

Timeframe/cohort entry

Any HCW recorded as being a “current” member of staff not on maternity leave (or similar) on the SWISS/GPCD databases on or after 1st March 2020 to date.

We will analyse separately those staff working solely in child health. For the purpose of excluding this group from the set of NHS staff working with adults, we defined a child health HCW as one who (i) is in a paediatric medical specialty (child and adolescent psychiatry, community child health, paediatric and perinatal pathology, paediatric cardiology, paediatric dentistry, paediatric surgery, paediatrics) or subspecialty (neonatal medicine), a child-focussed Agenda for Change (AFC) job family (Appendix 1), and/or (ii) is identified as working exclusively with people aged 18 or younger. For estimating the risk of COVID19 in child health healthcare workers, we will use a narrower definition restricted to criteria (i).

We will also analyse staff working in dentistry separately. We will do so for two reasons. First, there have been large changes in dental practice as a result of the pandemic with only emergency dental care taking place and secondly it is difficult to identify different specialties within dentistry using the SWISS database. Finally, we will also examine GPs separately (there are no data on staff members directly employed by practices other than GPs), since this group primarily work in the community rather than in hospital.

Basis of definitions

The following definitions (roles, occupations and grades) were refined after producing summary statistics of the numbers in each group, but BEFORE linking the SWISS database (the GPCD database had already been linked) to the databases containing the outcome data.

For the purposes of this analysis, likely roles have been defined narrowly. We aimed to identify those staff who, despite re-deployment as part of the COVID-19 response, are most likely to be in these roles. **This means that many staff who are patient-facing, and who even see patients with suspected COVID19 in “front-door” settings, may not be so classified**. For example, doctors in the “Clinical Pharmacology & Therapeutics” specialty frequently provide a general internal medicine service. However, where these doctors do not have “General (Internal) Medicine” (or similar) listed among their specialties they will *not* be classified as “Patient facing, front-door COVID”. We did so to avoid non-differential misclassification bias, which would lead us to under-estimate risk in these exposure groups. Nonetheless, these risks once estimated may be applied to members of staff fulfilling functions typically associated with these roles, regardless of their official job title, based on local knowledge.

Roles

Mutually exclusive and exhaustive roles were defined for all staff. Broad roles were “Undetermined”, “Non-patient facing” and “Patient facing” (Table 2a). Within “Patient facing” the following narrower roles were defined – “front-door COVID”, “Resp-oral-nasal-aerosol generating procedure prone role (AGP)”, “Intensive care” and “Patient facing, other” (Table 2b).

Staff of a seniority grade which implied that they were most likely to work in predominantly managerial/administrative areas were defined as non-patient facing. Staff who are senior managers

will therefore not be defined as “patient facing” regardless of their specialty or AFC job family. Nursing and midwifery staff who are band 8a were also assigned to “undetermined” with those in 8b-8d assigned non-patient-facing. Staff based exclusively in selected non-territorial Health Boards (Healthcare Improvement Scotland, NHS 24, NHS National Services Scotland (NEED TO CHECK IF STAFF PROVIDING SPECIALIST CLINICAL SERVICES – EG THE ADULT CYSTIC FIBROSIS SERVICE, ARE LISTED AS NSS STAFF - https://www.nss.nhs.scot/browse/specialist-healthcare/specialist-services) and NHS Education for Scotland) were also designated as non-patient facing. Other than this, roles were defined on the basis of specialties for medical and dental staff and AFC job families for other staff. For some staff information on their service area was also included (Tables 1a and 1b).

Table 1a Broad role

Definition	Description	Definition	Comparator(s)
Any staff	Any member of NHS staff.	Included in SWISS or GPCD database	General population
Patient facing, any	Include if likely to currently be working in patient-facing role.	Specific list of AFC roles and/or medical specialties (see Appendix 1)	General population
Non-patient-facing	Any member of NHS staff likely to be in a non-patient-facing role.	Specific list of AFC roles and/or medical specialties (see Appendix 1)	General population
Undetermined	Staff where it is not possible to allocate with confidence to patient-facing or non-patient-facing roles	Any staff not in “Patient-facing, any” or “Non-patient-facing” (see Appendix 1)	General population

Table 1b Narrow role (all are defined within patient-facing)

Definition	Description	Definition*	Comparator(s)
Patient-facing, front-door COVID19	Include if involved in acute medical receiving of patients with possible or probable COVID19 (ie not incidental finding such as COVID19 in patient with myocardial infarction).	Specific list of AFC roles and/or medical specialties and/or designated service area (see Appendix 1).	Patient-facing, other
Patient-facing, Resp-oral-nasal-AGP	Include if involved in work with high risk of exposure to oral, nasal or respiratory secretions and/or aerosol generating procedures (AGP).	Specific list of AFC roles and/or medical specialties and/or designated service area (see Appendix 1).	Patient-facing, other
Patient-facing, Intensive care	Intensive care medicine and anaesthetic specialties	Specific list of AFC roles and/or medical specialties and/or designated service area (see Appendix 1).	Patient-facing, other
Patient-facing, other	Patient-facing role but not front-door COVID or resp-oral-nasal-AGP or intensive care.	Patient-facing role but not front-door COVID or resp-oral-nasal-AGP or intensive care.	

*Nursing staff in the General Acute Nursing, Specialist Nursing and Bank Nursing AFC sub-job families, have been further assigned to specific roles according to the recorded service area (Appendix 1). This was done for the following territorial Health Boards, in whom there is >= 95% completeness for the service area variable: - NHS Ayrshire & Arran, NHS Borders, NHS Dumfries & Galloway, NHS Forth Valley, NHS Grampian, NHS Greater Glasgow & Clyde, NHS Highland, NHS Orkney and NHS Shetland. The remaining territorial boards had lower completeness for service area (ranging from 91% to <1%)

Covariates

In addition to age and sex, the covariates shown in Table 3 were defined.

Table 2 Covariates

Name	Definition
SWISS only	
Immigration status	Staff having any immigration status (ie being a non-national) recorded on SWISS yes/no.
Any long-term illness	Defined using self-described "Longstanding illness"
Length of service	Length of service in years
Household composition	Number of individuals in household
SWISS and general population	
Chronic diseases	Pre-specified definitions based on SMR01 and pseudo-BNF codes. These are ischaemic heart disease, other heart disease, other circulatory disease, chronic kidney disease, chronic lower respiratory disease, neurological disorders, liver disease, immunodeficiency and immunosuppression, neoplasm, disorders of oesophagus, stomach and duodenum (see Appendix

	for definitions).
Ethnicity	ONOMAP-derived ethnicity (https://www.onomap.org/).
Scottish index of multiple deprivation	Area based measure of socio-economic deprivation

Note that ethnicity is also recorded in the SWISS database (https://www.isdscotland.org/Health-Topics/Workforce/NES-Publication/Equality_and_Diversity_S2019.xlsx) but this is not yet available in data available to PHS hence we will use general population definitions. Moreover, even when available there are high levels of missingness for ethnicity, with around 19.4% responding "not known" and 12% declining to answer.

We will also define each staff member's grade. For medical and dental job family we collapsed the grade and medical grade variables into three broad categories; consultant, specialty and associate specialist and "training" grades. The latter category contains a broad range of roles. For the remaining job families, staff were grouped into grades 1-4, grades 5-7, and grade 8 or above.

Based on AFC job family, staff will also be allocated into broadly defined occupations, which are shown in Table 3.

Table 3 Occupations

Agenda for Change Job Family	Number of staff members
Nursing and Midwifery	69499
Administrative Services	29522
Support Services	17708
Medical and Dental	15342
Allied Health Profession	14251
Other (includes Ambulance Services, Dental Support, Healthcare Sciences, Medical Support, Other Therapeutic, Personal And Social Care, Senior Managers, Unallocated / Not Known)	20625
General practice – GP partners and salaried GPs *only*	5400

Outcomes

We will use selected outcome definitions from the COVID19 case-control study (REACT) (Table 4). OF these, "B Test positive and hospitalised" is the primary outcome for this analysis.

Table 4 also shows the event counts for these outcomes as of the 15th of May 2020, for the whole of Scottish population including the working age (25 to 64 years) and non-working age population.

Table 4 Selected COVID-19 outcomes for the entire Scottish population, as well as counts of these events, for the working age and non-working age populations

Outcome	Working age	Non-working age
A Test positive for SARS-COV2	7395	7669
B Test positive and hospitalised	1864	3433
C Test positive and ICU	389	235
D Test positive, no ICU and died	389	235

E Test positive and ICU or died	591	3201
F Test negative or no test, COVID19 on NRS death	106	1180
Population denominator	3000000	2500000

Statistical analysis

Sample sizes and event numbers

On applying the population-wide working age rates to the SWISS/GPCD samples, assuming the rates are the same in HCWs as in the general population, the expected counts shown in Table 5 are obtained.

Table 5 Rate per 1000 for working age population and expected counts for each professional grouping

Outcome	Rate per 1000	Nursing and Midwifery	Administrative Services	Support Services	Medical and Dental	Allied Health Profession	Other groups	GP	**Total**
A Test positive	2.46	170.97	72.62	43.56	37.74	35.06	50.74	12.4	425.55
**B Test positive and hospitalised**	**0.62**	**43.09**	**18.3**	**10.98**	**9.51**	**8.84**	**12.79**	**3.13**	**107.26**
C Test positive and ICU	0.13	9.03	3.84	2.3	1.99	1.85	2.68	0.66	22.48
D Test positive, no ICU and died	0.13	9.03	3.84	2.3	1.99	1.85	2.68	0.66	22.48
E Test positive and ICU or died	0.2	13.9	5.9	3.54	3.07	2.85	4.13	1.01	34.6
F Test negative or no test, COVID19 on NRS death	0.04	1.18	0.57	0.202	0.614	2.78	0.825	0.708	6.92

We will present baseline characteristics as well as counts of events (and rates) for staff who had any test, as well as for the above outcomes. We will present counts and rates for COVID-19 hospitalisation, intensive care unit admission and death for staff members themselves, as well as for their households. We will present these summary statistics according to the broad (Table 2a), and narrow roles (Table 2b), occupations (Table 3) and seniority grades.

Comparison of HCWs with general population

We will compare rates in each of the broad role definitions – “patient facing”, “non-patient facing” and “undetermined” (Table 2a) to the general population via linkage to the existing case-control REACT study. We will estimate rate ratios using conditional logistic regression models which allow

for the matching on age, sex and general practice area used in the case-control design. In further models we will additionally adjust for the covariates listed in Table 2.

Comparison within HCWs

Within the HCW cohort, we will compare rates across the narrow role definitions – “front-door COVID”, “Resp-oral-nasal-AGP”, “Intensive care” and “Patient facing, other” (Table 2b) using Cox regression models. We will fit the model using calendar time, treating 1st March 2020 as day 1. We will stratify into four groups of Health Boards:- Greater Glasgow and Clyde; Lanarkshire; Ayrshire and Arran, Borders, Dumfries and Galloway, Fife, Forth Valley, Lothian, and Tayside; Grampian, Highland, Orkney, Shetland, Western Isles. The groups were chosen because on plotting the cumulative incidence of COVID-19 hospitalisation over time for the whole Scottish population, these boards were similar. The groups also make sense in terms of the geography of Scotland. We will initially adjust for age and sex, and will subsequently add terms for the covariates listed in Table 3. Finally, we will include terms for occupation (Table 3) and grade. As occupation and grade are of interest per se (not just as potential confounders) we will present hazard ratios for the effect of each of these in combination (by additional relevant coefficients on the linear predictor scale). We will test for interactions between specific exposure groups, although such tests are expected to lack statistical power in view of the comparatively small numbers of events expected (Table 5). Some households include more than one member of staff. To accommodate this, we will specify household as a clustering variable within the survival::coxph function, which allows for correlation in the calculation of valid standard errors in the presence of clustering.

Household members

We will repeat the modelling described above, but with hospitalisation for COVID-19 in a household member as the outcome variable. We will restrict the household member analysis to households with 6 or fewer individuals to avoid including staff residing in institutions.

Presenting risk for HCWs

Using outputs from the above models (and the baseline hazards), we will present estimates of the risk of COVID-19 hospitalisation at the censoring date, alongside 95% confidence intervals. These risks will be presented according to age, sex, role, comorbidity and any other influential variables identified in the modelling.

Review

Within the PHS network, much of the R code for the preceding analyses as well as some summary statistics and tables can be viewed here (https://phs-git.nhsnss.scot.nhs.uk/COVID-19/health_care_workers).

Appendix 1 List of specialties/job roles/service areas

Medical specialties

	Non patient-facing	Patient-facing			
		Any	Front-door	Resp-oral-nasal-AGP	Intensive care
Acute Internal Medicine		Yes	Yes		
Allergy					
Anaesthetics		Yes			Yes
Audio Vestibular Medicine					
Audiological Medicine					
Blood Transfusion					
Breast Screening Service					
Cardiology		Yes			
Cardiothoracic Surgery		Yes			
Chemical Pathology					
Child And Adolescent Psychiatry					
Clinical Genetics					
Clinical Neurophysiology					
Clinical Oncology		Yes			
Clinical Pharmacology and Therapeutics		Yes			
Clinical Radiology					
Community Child Health					
Community Dentistry		Yes		Yes	
Community Psychiatry		Yes			
Community Sexual And Reproductive Health		Yes			
Dental & Maxillofacial Radiology					
Dental Public Health					
Dermatology					
Diagnostic Neuropathology	Yes				
Emergency Medicine		Yes	Yes		
Endocrinology and Diabetes		Yes			
Endodontics		Yes		Yes	
Ent Surgery		Yes		Yes	
Family Planning Service					
Fixed & Removable Prosthodontics					
Forensic Histopathology	Yes				
Forensic Psychiatry					
Gastroenterology		Yes			

General (Internal) Medicine		Yes	Yes		
General Psychiatry		Yes			
General Surgery		Yes			
Genito-Urinary Medicine		Yes			
Geriatric Medicine		Yes	Yes		
GP Other Than Obstetrics		Yes			
Haematology					
Histopathology	Yes				
Homeopathy					
Immunology					
Infectious Diseases		Yes	Yes		
Intensive Care Medicine		Yes			Yes
Medical Microbiology And Virology					
Medical Oncology		Yes			
Medical Ophthalmology		Yes			
Microbiology					
Neurology		Yes			
Neurosurgery		Yes			
Nuclear Medicine					
Obstetrics And Gynaecology		Yes			
Occupational Medicine		Yes			
Old Age Psychiatry		Yes			
Ophthalmology		Yes			
Oral And Maxillofacial Surgery		Yes		Yes	
Oral And Maxillofacial Pathology	Yes				
Oral Medicine		Yes		Yes	
Oral Microbiology					
Oral Pathology					
Oral Surgery		Yes		Yes	
Orthodontics		Yes		Yes	
Otolaryngology		Yes		Yes	
Paediatric And Perinatal Pathology	Yes				
Paediatric Cardiology		Yes			
Paediatric Dentistry		Yes		Yes	
Paediatric Surgery		Yes			
Paediatrics		Yes	Yes		
Pain Management					
Palliative Medicine					
Plastic Surgery		Yes			
Psychiatry Of Learning Disability					
Psychotherapy					
Public Health Medicine	Yes				
Rehabilitation Medicine		Yes			
Renal Medicine		Yes			

Respiratory Medicine		Yes		Yes	
Restorative Dentistry					
Rheumatology		Yes			
Special Care Dentistry		Yes		Yes	
Surgical Dentistry		Yes		Yes	
Trauma And Orthopaedic Surgery		Yes			
Urology		Yes			
Vascular Surgery		Yes			
Virology					

There are two specialty fields in the SWISS database. These are “specialty” with around 85% completeness, and “second specialty” with lower completeness. We added data from TURAS People (which holds data on doctors in training roles) to increase the completeness to approximately 98%.

Agenda for change job families

		Non patient-facing	Patient facing			
Job Family	Job Sub Family		Any	Front-door	Resp-oral-nasal-AGP	Job Family
ADMINISTRATIVE SERVICES	FINANCE	Yes				
ADMINISTRATIVE SERVICES	HUMAN RESOURCES	Yes				
ADMINISTRATIVE SERVICES	INFORMATION SYSTEMS/TECHNOLOGY	Yes				
ADMINISTRATIVE SERVICES	NA	Yes				
ADMINISTRATIVE SERVICES	NHS24 CALL HANDLER	Yes				
ADMINISTRATIVE SERVICES	OFFICE SERVICES	Yes				
ADMINISTRATIVE SERVICES	PATIENT SERVICES	Yes				
ALLIED HEALTH PROFESSION	AHP TRAINING/ADMINISTRATION	Yes				
ALLIED HEALTH PROFESSION	AMBULANCE PARAMEDIC		Yes	Yes		
ALLIED HEALTH PROFESSION	ARTS THERAPIES					
ALLIED HEALTH PROFESSION	DIAGNOSTIC RADIOGRAPHY		Yes			
ALLIED HEALTH PROFESSION	DIETETICS					
ALLIED HEALTH PROFESSION	GENERIC THERAPIES					
ALLIED HEALTH PROFESSION	OCCUPATIONAL THERAPY		Yes			
ALLIED HEALTH PROFESSION	ORTHOPTICS					
ALLIED HEALTH PROFESSION	ORTHOTICS		Yes			
ALLIED HEALTH PROFESSION	PHYSIOTHERAPY		Yes		Yes	
ALLIED HEALTH PROFESSION	PODIATRY		Yes			
ALLIED HEALTH PROFESSION	PROSTHETICS					
ALLIED HEALTH PROFESSION	SPEECH AND LANGUAGE THERAPY		Yes			
ALLIED HEALTH PROFESSION	THERAPEUTIC RADIOGRAPHY		Yes	Yes		

AMBULANCE SERVICES	AMBULANCE CARE ASSISTANT		Yes	Yes		
AMBULANCE SERVICES	AMBULANCE TECHNICIAN		Yes	Yes		
AMBULANCE SERVICES	DRIVER		Yes	Yes		
AMBULANCE SERVICES	EMDC OPERATIVE	Yes				
AMBULANCE SERVICES	OPERATIONAL MANAGER	Yes				
AMBULANCE SERVICES	PTS DAY CONTROL	Yes				
DENTAL SUPPORT	CLINICAL DENTAL TECHNICIAN		Yes		Yes	
DENTAL SUPPORT	DENTAL HYGIENIST		Yes		Yes	
DENTAL SUPPORT	DENTAL HYGIENIST-THERAPIST		Yes		Yes	
DENTAL SUPPORT	DENTAL NURSING		Yes		Yes	
DENTAL SUPPORT	DENTAL TECHNICIAN		Yes		Yes	
DENTAL SUPPORT	DENTAL THERAPIST		Yes		Yes	
DENTAL SUPPORT	ORAL HEALTH					
DENTAL SUPPORT	ORTHODONTIC THERAPIST		Yes		Yes	
EMERGENCY SERVICES	AMBULANCE AUXILIARY		Yes	Yes		
EMERGENCY SERVICES	AMBULANCE CARE ASSISTANT		Yes	Yes		
EMERGENCY SERVICES	AMBULANCE PARAMEDIC		Yes	Yes		
EMERGENCY SERVICES	AMBULANCE TECHNICIAN		Yes	Yes		
EMERGENCY SERVICES	DRIVER		Yes	Yes		
EMERGENCY SERVICES	EMDC OPERATIVE	Yes				
EMERGENCY SERVICES	OPERATIONAL MANAGER	Yes				
EMERGENCY SERVICES	PTS DAY CONTROL					
HEALTHCARE SCIENCES	BIOMEDICAL SCIENCES LIFE					
HEALTHCARE SCIENCES	CLIN PHOTO/ILLUSTRATE PHYSICAL					
HEALTHCARE SCIENCES	CLINICAL PERFUSION PHYSIOLOGY					
HEALTHCARE SCIENCES	CLINICAL PHYSIOLOGY					
HEALTHCARE SCIENCES	CLINICAL SCIENCES LIFE					
HEALTHCARE SCIENCES	CLINICAL SCIENCES PHYSICAL					

HEALTHCARE SCIENCES	CLINICAL SCIENCES PHYSIOLOGY					
HEALTHCARE SCIENCES	CLINICAL TECHNOLOGY LIFE					
HEALTHCARE SCIENCES	CLINICAL TECHNOLOGY PHYSICAL					
HEALTHCARE SCIENCES	MAXILLOFACIAL PROS PHYSICAL					
HEALTHCARE SCIENCES	NA					
HEALTHCARE SCIENCES	STERILE SERVICES LIFE					
MEDICAL AND DENTAL SUPPORT	DENTAL NURSING		Yes		Yes	
MEDICAL AND DENTAL SUPPORT	DENTAL TECHNOLOGY		Yes		Yes	
MEDICAL AND DENTAL SUPPORT	OPERATING DEPARTMENT		Yes			
MEDICAL AND DENTAL SUPPORT	ORAL HEALTH		Yes		Yes	
MEDICAL AND DENTAL SUPPORT	PHYSICIANS ASSISTANT					
MEDICAL AND DENTAL SUPPORT	THEATRE SERVICES		Yes			
MEDICAL SUPPORT	OPERATING DEPARTMENT		Yes			
MEDICAL SUPPORT	PHYSICIANS ASSISTANT		Yes			
MEDICAL SUPPORT	THEATRE SERVICES		Yes			
NURSING AND MIDWIFERY	COMMUNITY CHILDREN'S NURSING					
NURSING AND MIDWIFERY	MIDWIFERY DIRECT CC		Yes			
NURSING AND MIDWIFERY	MIDWIFERY INDIRECT CC		Yes			
NURSING AND MIDWIFERY	NA					
NURSING AND MIDWIFERY	NEONATAL MIDWIFERY CC		Yes			
NURSING AND MIDWIFERY	NEONATAL MIDWIFERY DIRECT CC		Yes			
NURSING AND MIDWIFERY	NEONATAL MIDWIFERY INDIRECT CC		Yes			
NURSING AND MIDWIFERY	NEONATAL NURSING DIRECT CC		Yes			
NURSING AND MIDWIFERY	NEONATAL NURSING INDIRECT CC		Yes			
NURSING AND MIDWIFERY	NHS 24 NURSING	Yes				
NURSING AND MIDWIFERY	NURSING TRAINING/ADMIN/MGT					
NURSING AND	PAEDIATRIC NURSING		Yes	Yes		

MIDWIFERY						
NURSING AND MIDWIFERY	PRACTICE NURSING		Yes			
NURSING AND MIDWIFERY	PUBLIC HEALTH NURSING					
NURSING AND MIDWIFERY	SCHOOL NURSING					
NURSING AND MIDWIFERY	SEXUAL AND REPRODUCTIVE HEALTH					
NURSING AND MIDWIFERY	SPECIALIST NURSING		Yes			
NURSING AND MIDWIFERY	STAFF NURSERY					
NURSING AND MIDWIFERY	TREATMENT ROOM NURSING		Yes			
NURSING AND MIDWIFERY	BANK NURSING		Yes			
NURSING AND MIDWIFERY	BTS NURSING		Yes	Yes		
NURSING AND MIDWIFERY	CARE OF THE ELDERLY NURSING		Yes	Yes		
NURSING AND MIDWIFERY	COMMUNITY GENERAL NURSING		Yes			
NURSING AND MIDWIFERY	DISTRICT NURSING		Yes			
NURSING AND MIDWIFERY	FAMILY PLANNING NURSING					
NURSING AND MIDWIFERY	GENERAL ACUTE NURSING		Yes	Yes		
NURSING AND MIDWIFERY	HEALTH VISITOR NURSING					
NURSING AND MIDWIFERY	LEARNING DISABILITIES NURSING		Yes			
NURSING AND MIDWIFERY	MENTAL HEALTH NURSING		Yes			
NURSING AND MIDWIFERY	MIDWIFERY		Yes			
OTHER THERAPEUTIC	GENETIC COUNSELLING					
OTHER THERAPEUTIC	NA					
OTHER THERAPEUTIC	OPTOMETRY					
OTHER THERAPEUTIC	PHARMACY					
OTHER THERAPEUTIC	PHARMACY TECHNICIANS	Yes				
OTHER THERAPEUTIC	PLAY SPECIALIST		Yes			
OTHER THERAPEUTIC	PSYCHOLOGY					
PERSONAL AND SOCIAL CARE	CARE AT HOME		Yes			
PERSONAL AND SOCIAL CARE	HEALTH PROMOTION					
PERSONAL AND SOCIAL CARE	HOSPITAL CHAPLAINCY					

PERSONAL AND SOCIAL CARE	RESIDENTIAL / DAY CARE		Yes			
PERSONAL AND SOCIAL CARE	SOCIAL WORK					
SENIOR MANAGERS	NA	Yes				
SUPPORT SERVICES	CATERING SERVICES					
SUPPORT SERVICES	DOMESTIC SERVICES					
SUPPORT SERVICES	ESTATES					
SUPPORT SERVICES	GENERAL SERVICES					
SUPPORT SERVICES	GROUNDS SERVICES					
SUPPORT SERVICES	HOTEL SERVICES					
SUPPORT SERVICES	LAUNDRY/LINEN SERVICES					
SUPPORT SERVICES	NA					
SUPPORT SERVICES	PORTERING SERVICES		Yes			
SUPPORT SERVICES	SECURITY SERVICES					
SUPPORT SERVICES	STERILE SERVICES					
SUPPORT SERVICES	STORES SERVICES					
SUPPORT SERVICES	TRANSPORT SERVICES					
UNALLOCATED / NOT KNOWN	NA					
UNALLOCATED / NOT KNOWN	NOT KNOWN					

Clinical nurse specialist Do not use for definitions

Retain description for information, but these definitions are under review. As such, the data in SWISS will not reflect accurate CNS staffing "on the ground" and these definitions should not be included in the project.

**Clinical Nurse Speciality (CNS)**	**Patient-facing**	**Front-door**	**Resp-oral-nasal-AGP**
Addictions			
Alcohol			
Anti-coagulant			
Asthma	Yes		
Blood Transfusion	Yes		
Breast Care Nursing	Yes		
Breast Feeding Co-ordinator			
Burns	Yes		
Cancer	Yes		
Cancer – Breast	Yes		
Cancer – Chemotherapy	Yes		
Cancer – Colorectal	Yes		
Cancer - Gynaecology Oncology	Yes		
Cancer – Haematology	Yes		
Cancer - Head and Neck	Yes		
Cancer – Lung	Yes		
Cancer - Neuro-oncology	Yes		

Cancer – Oncology	Yes		
Cancer – Ophthalmic	Yes		
Cancer – Other	Yes		
Cancer - Radiotherapy and Oncology	Yes		
Cancer – Sarcoma	Yes		
Cancer – Skin	Yes		
Cancer - Upper GI	Yes		
Cancer - Urology Oncology	Yes		
Cardiac Rehabilitation			
Cardiac Surgery	Yes		
Cardiology	Yes		
Challenging Behaviour			
Child and Adolescent Mental Health			
Child Protection			
Cognitive Behavioural Therapy			
Continence			
Cystic Fibrosis	Yes		Yes
Deliberate Self Harm			
Dermatology			
Diabetes	Yes		
Drugs			
Ear Nose and Throat	Yes		Yes
Emergency Medicine	Yes	Yes	
Endocrinology	Yes		
Epilepsy	Yes		
Forensics			
Gastro-intestinal	Yes		
Genetic			
Genitourinary Medicine	Yes		
Gynaecology	Yes		
Haematology	Yes		
HIV			
Hospital at Night	Yes		
Infection Control	Yes		
Mental Illness			
Midwifery	Yes		
Midwifery - Fetal Medicine	Yes		
Midwifery - Special Needs in Medicine	Yes		
Minor Injuries	Yes		
Multiple Sclerosis	Yes		
Neonatology	Yes		
Neuroscience/Neurology	Yes		
Nutrition			
Occupational Health and Safety	Yes		

Older Peoples Care / Gerontology	Yes		
Ophthalmic			
Orthopaedics	Yes		
Paediatric	Yes		
Pain			
Palliative	Yes		
Parkinson's Disease	Yes		
Perinatal Mental Health			
Plastic Surgery	Yes		
Renal	Yes		
Respiratory	Yes		Yes
Resuscitation			
Rheumatology	Yes		
Severe and Enduring Illness			
Sexual Health/Family Planning			
Smoking Cessation			
Stoma			
Stroke	Yes		
Substance Misuse			
Tissue Viability			
Urological	Yes		
Vascular	Yes		
Other - please specify			

Service areas

service_area	Front-door	Resp-oral-nasal-AGP	Intensive care
Accident and Emergency	Yes		
Anaesthetics			Yes
Anatomical Pathology			
Audiological Medicine			
Bank Management			
Biochemistry			
Biomedical Science			
Breast Screening			
Cancer			
Cardiac			
Clinical Audit			
Clinical Genetics			
Clinical Oncology			
Clinical Pathology			
Clinical Pharmacology & Therapeutics			
Clinical Radiology (Diagnostic)			

Combination			
Communications			
Counselling			
Criminal Justice/Police Custody			
Dermatology			
Ear Nose & Throat		Yes	
Endocrinology & Diabetes			
Facilities			
Family Planning Service			
Fixed And Removable Prosthodontics			
Forensic Psychiatry			
Gastroenterology			
General Dental Practice		Yes	
General Medicine	Yes		
General Practice			
General Psychiatry			
General Surgery			
Genito-Urinary Medicine			
Gynaecology			
Haematology			
Health and Safety			
Health Promotion			
Health Protection Nurses			
Histopathology			
Homeless Services			
Homeopathy			
Hospital at Night			
Immunology			
Infection Control			
Infectious Diseases	Yes		
Intensive Care			Yes
Keep Well Type Initiatives			
Learning Disabilities			
Medical Engineering			
Medical Illustration			
Medical Microbiology & Virology			
Medical Records			
Microbiology			
Midwifery Direct Clinical Care			
Midwifery Indirect Clinical Care			
NA			
Neonatal Midwifery Direct Clinical Care			
Neonatal Midwifery Indirect Clinical Care			
Neonatal Nursing Direct Clinical Care			

Neonatal Nursing Indirect Clinical Care			
Neonatal/SCBU			Yes
Neuroscience			
Nuclear Medicine			
Obstetrics			
Occupational Medicine			
Ophthalmology			
Oral & Maxillofacial		Yes	
Oral Medicine			
Oral Surgery			
Orthodontics			
Out of Hours			
Pain Management			
Pathology			
Payroll			
Pharmacy			
Phlebotomy			
Physiology			
Planning			
Plastic Surgery			
Psychotherapy			
Public Health Medicine			
Radiology			
Rehabilitation			
Renal			
Reproductive			
Research and Development			
Respiratory		Yes	
Restorative Dentistry		Yes	
Rheumatology			
Sexual Health			
Shops and Services			
Smoking Cessation			
Social Care			
Specialist Public Health Initiatives			
Stroke			
Surgical Dentistry		Yes	
Theatre Services			
Training			
Trauma & Orthopaedic Surgery			
Urology			
Vascular Surgery			
Young Peoples Nurse/Healthy Lifestyle Focused			

Appendix 2 – Notes on GPCD CHI seeding and household linkage

The following need to be considered when analysing households.

Household was generally estimated using the address as supplied in CHI. However, where the number of household members was above 6 we additionally defined households using a fuzzy match (ie allowing for some differences in spelling and capitalisation) on surname. The result of this additional criterion is that some GPs will be recorded as belonging to single person households when in fact they live in multi-person households.

3 records have no postcode recorded on the CHI database. They have been included along with their households as they match well enough solely on names and address text.

Many records have households where some or all members have hyphenated surnames (ordering and completeness variable). Other householders had just one element (or vice versa) of the hyphenated name, this group also contained a large number of non-residential addresses recorded on the CHI file (eg GP practices where many people have addresses recorded) resulting in large households. This was addressed by fuzzy matching surname and checking address move dates. A small number of GPs in this analysis appear to have their address listed as the practice address.

Appendix 3 – Notes on TURAS/SWISS

- For doctors in training their Health Board is recorded as NHS Education Scotland. Therefore, NHS Education Scotland applies an algorithm to determine the Health Board of placement. This derived variable is supplied to PHS for all subsequent analysis. Similarly, where the specialty from doctors in training is available on the TURAS system it is taken from there. Otherwise it is taken from SWISS.
- Multiple serial monthly extracts are taken from SWISS and supplied to NES. These were joined to allow identification of all staff working for the NHS over the time period of interest.
- Ethnicity and “long-term condition” are “equality and diversity” fields not normally supplied to NHS Education Scotland as part of the database extract and so may need to be added later
- Some values in the agenda for change job family/sub-job family fields are rarely completed and so the apparent level of granularity from the data dictionary does not always reflect the true granularity
- NHS Education Scotland run a number of analyses to clean and transform the database for their own reports. These were applied prior to transfer of the data to PHS

Appendix 4 – Data dictionary

This includes data items not defined elsewhere (eg on the SWISS or SMR01 manuals)

GPCD

- HID is household level ID
- LEAD is the GP from the original file. (0 means household member)
- SERIAL_NO is the original key from the GPCD data

Appendix 5 – Note on testing

As part of the COVID-19 response, the UK government set-up Lighthouse lab which are non-NHS labs set-up rapidly in response to a need to increase testing capacity staffed from universities and industry. The results from these labs are not currently visible within NHS or Public Health Scotland systems. However, the policy in Scotland is that health and social care workers who require testing to clarify if they can return to work should always have had – and will continue to have – their tests processed by an NHS lab, NOT in a Lighthouse lab. To date, the Lighthouse lab has been used for other key workers, lower down the prioritisation matrix (not level 1a or 1b))
https://www.gov.scot/publications/coronavirus-covid-19-getting-tested/pages/overview/

Appendix 6 – Definitions of comorbidities

The following R code shows how each comorbidity were defined.

```
################  IHD ####################################

## nitrates are BNF code 020601

ids.icd.IHD <- unique(diagnoses$ANON_ID[grep("^I2[0-5]", diagnoses$ICD10)])

ids.bnf.IHD <- unique(scrips$ANON_ID[substr(as.character(scrips$bnf_paragraph_code), 1, 6) == "020601" |

                          substr(as.character(scrips$bnf_paragraph_code), 1, 6) == "020603"])

table(ids.bnf.IHD %in% ids.icd.IHD)

## procedure codes for CABG and PTCA

ids.procedures.IHD <- unique(procedures$ANON_ID[grep("^K4[012349]|^K50", procedures$MAIN_OPERATION)])

ids.IHD <- unique(c(ids.icd.IHD, ids.bnf.IHD, ids.procedures.IHD))

cc.severe$IHD.any <- as.factor(as.integer(cc.severe$ANON_ID %in% ids.IHD))

##### other heart disease ####################################

## heart disease is I05 to I52

ids.icd.heart.other <- unique(diagnoses$ANON_ID[grep("^I0[01256789]|^I1[0-5]|^I2[6-8]|^I3[0-9]|^I4[0-9]|^I5[0-2]",

                        diagnoses$ICD10)])

ids.bnf.heart.other <-

   unique(scrips$ANON_ID[substr(as.character(scrips$bnf_paragraph_code), 1, 4) == "0203"])  # anti-arrhythmics

table(ids.bnf.heart.other %in% ids.icd.heart.other)

ids.procedures.heart.other <- unique(procedures$ANON_ID[grep("^K57", procedures$MAIN_OPERATION)])

ids.heart.other <- unique(c(ids.icd.heart.other, ids.bnf.heart.other, ids.procedures.heart.other))

cc.severe$heart.other.any <- as.factor(as.integer(cc.severe$ANON_ID %in% ids.heart.other))

############ other circulatory disease is I60 to I99

ids.icd.circulatory.other <-

   unique(diagnoses$ANON_ID[grep("^I[6-9]|^Z95", diagnoses$ICD10)])

cc.severe$circulatory.other <-

   as.factor(as.integer(cc.severe$ANON_ID %in% ids.icd.circulatory.other))

############# chronic kidney disease ##########################

## this includes CKD stage 4

ids.icd.ckd <- unique(diagnoses$ANON_ID[grep("^N18[45]|^Z49[0-2]|^Z94[02]",

                            diagnoses$ICD10)])
```

```
ids.kidneytransplant <- unique(procedures$ANON_ID[grep("^M01[1234589]",
                                        procedures$MAIN_OPERATION)])
table(ids.kidneytransplant %in% ids.icd.ckd)
ids.ckd.any <- unique(c(ids.icd.ckd, ids.kidneytransplant))
cc.severe$ckd.any <-  as.factor(as.integer(cc.severe$ANON_ID %in% ids.ckd.any))
##### asthma and chronic lower respiratory disease #################
ids.icd.asthma <- unique(diagnoses$ANON_ID[grep("^J4[56]", diagnoses$ICD10)])
ids.icd.chronresp <- unique(diagnoses$ANON_ID[grep("^J4[012347]|^J6[0-9]|^J70|^J8[0-6]|^J9[0-9]|^G47\\.?3",
                                      diagnoses$ICD10)])
ids.bnf.broncho <- unique(scrips$ANON_ID[as.integer(scrips$sectioncode) >= 301 &
                              as.integer(scrips$sectioncode) <= 303])
table(ids.icd.asthma %in% ids.bnf.broncho)
table(ids.icd.chronresp %in% ids.bnf.broncho)
ids.oad.any <- unique(c(ids.icd.asthma, ids.icd.chronresp, ids.bnf.broncho))
cc.severe$oad.any <- as.factor(as.integer(cc.severe$ANON_ID %in% ids.oad.any))
##### Neurological disorders #######################
## include all Nervous chapter except G40 "Episodic and Paroxysmal Disorders"
## Except G0 meningitis and encephalitis, and G5 local neuropathies
## also include F03 dementia NOS
ids.icd.neuro <- unique(diagnoses$ANON_ID[grep("^F03|^G[1236789]", diagnoses$ICD10)])
ids.bnf.neuro <- unique(scrips$ANON_ID[as.integer(scrips$sectioncode) == 409 |
                              as.integer(scrips$sectioncode) == 411])
table(ids.bnf.neuro %in% ids.icd.neuro)
## these drugs listed by HPS pharmacist as used for multiple sclerosis
## interferon beta 080204M, Glatiramer acetate 0802040U0, Natalizumab 0802040W0
## Dimethyl fumar 0802040AK, Teriflunomide 0802040AL, Alemtuzumab 0802030
## no records in scrips for these drugs
## 526 records in scrips[substr(scrips$bnf_paragraph_code, 1, 5) == "08020", ]
ids.neuro.any <- unique(c(ids.icd.neuro, ids.bnf.neuro))
cc.severe$neuro.any <- as.factor(as.integer(cc.severe$ANON_ID %in% ids.neuro.any))
############## Liver disease ##############################################
liver.grep.string <-
"^C22\\.?0|^I85\\.?0|^I98\\.?3|^K70\\.?[234|^K71\\.?7|^K72\\.?[019]|^K72\\.?[019|^K73|^K74\\.?[023456]|^K76\\.?7|^R18"
table(grep(liver.grep.string, diagnoses$ICD10, value=TRUE))
ids.icd.liver <- unique(diagnoses$ANON_ID[grep(liver.grep.string, diagnoses$ICD10)])
cc.severe$liver.any <- as.factor(as.integer(cc.severe$ANON_ID %in% ids.icd.liver))
#### Immunodeficiency and immunosuppression ##################################
## immune.any includes primary immunodeficiency and secondary immunosuppression
ids.icd.immune <- unique(diagnoses$ANON_ID[grep("^B2[0-3|^D8[0-9]", diagnoses$ICD10)])
## 802 other immunomodulating drugs
```

```
## Methotrexate and chloroquine appear in musculoskeletal chapter

ids.bnf.immune <- unique(scrips$ANON_ID[as.integer(scrips$sectioncode) == 802 |

                                as.integer(scrips$paracode) == 50301])

ids.immune.any <- unique(c(ids.icd.immune, ids.bnf.immune))

cc.severe$immune.any <- as.factor(as.integer(cc.severe$ANON_ID %in% ids.immune.any))

############# neoplasms ################

ids.icd.neoplasm <- unique(diagnoses$ANON_ID[grep("^C[0-9]|^D[0-4]", diagnoses$ICD10)])

ids.bnf.neoplasm <- unique(scrips$ANON_ID[as.integer(scrips$sectioncode) == 801])

ids.neoplasm.any <- unique(c(ids.icd.neoplasm, ids.bnf.neoplasm))

cc.severe$neoplasm.any <- as.factor(as.integer(cc.severe$ANON_ID %in% ids.neoplasm.any))

###### disorders of esophagus, stomach and duodenum ############################

ids.esoph.stomach.duod <-  unique(diagnoses$ANON_ID[grep("^K2[0-9]|^K3[01]", diagnoses$ICD10)])

cc.severe$esoph.stomach.duod <-

  as.factor(as.integer(cc.severe$ANON_ID %in% ids.esoph.stomach.duod))
```
